# Marine Carotenoids: A Critical Review of Bioactivities, Bioavailability, and Therapeutic Potential

**DOI:** 10.1155/bmri/4147524

**Published:** 2025-12-29

**Authors:** Gamal M. El-Sherbiny, Mohamed H. Kalaba

**Affiliations:** ^1^ Botany and Microbiology Department, Faculty of Science, Al-Azhar University, Nasr City, Cairo, Egypt, azhar.edu.eg

**Keywords:** *α*, *β*-carotene, astaxanthin, bioavailability, canthaxanthin, fucoxanthin, halocynthiaxanthin, lycopene, neoxanthin, peridinin, spirilloxanthin, zeaxanthin

## Abstract

The marine environment is a significant origin of bioactive substances like carotenoids. Marine carotenoids are secondary metabolites with mechanism‐anchored benefits across redox, immune, and metabolic pathways, comprising antioxidant, anti‐inflammatory, antidiabetic, anticancer, and antimicrobial activities. These bioactive compounds have garnered significant interest from the pharmaceutical, nutraceutical, and cosmetic industries, driving the exploration for novel natural reservoirs of carotenoids. However, most of the research has focused on carotenoids found in fruits, vegetables, and other higher plant components. Despite increasing interest, there are few publications on carotenoids found in marine sources such as seaweed, microalgae, and marine animals. This review summarizes chemistry, biosynthesis, extraction methods, bioavailability, and the bioactivities reported for major marine carotenoids (e.g., *α*‐ and *β*‐carotene, lycopene, fucoxanthin, astaxanthin, zeaxanthin, canthaxanthin, spirilloxanthin, halocynthiaxanthin, neoxanthin, and peridinin).

## 1. Overview of Carotenoids

Carotenoids are bioactive compounds that have demonstrated specific, mechanism‐linked benefits (e.g., singlet‐oxygen quenching, nuclear factor‐*κ*B (NF‐*κ*B) inhibition, Nrf2 activation, and modulation of lipid peroxidation). These naturally occurring pigments belong to a class of polyisoprenoids and are produced by algae, plants, cyanobacteria, fungi, and bacteria. They have yellow and orange–red pigments. Carotenoids are the greatest universal and varied category of dyes found in nature. There are more than 700 types of naturally existing carotenoids, and this quantity keeps growing as new ones are found. Depending on their chemical composition, carotenoids are classified into two main groups. First‐group carotenes are pure hydrocarbons that are oxygen‐free, like *α*‐ and *β*‐carotene and lycopene. The second group consists of xanthophylls, which comprise zeaxanthin, lutein, and cryptoxanthin. Xanthophylls are carotene derivatives that have been oxygenated and include one or more oxygen groups [1].

The marine environment is one of the most significant bioreservoirs explored, which is considered to be home to a variety of marine species with unique biological characteristics and has enormous potential as an origin of active components for the improvement of novel therapeutic drugs [2, 3]. Marine plants and animals, as well as phytoplankton, produce most of the marine carotenoids that are found in the ocean. Carotenoids, which are accountable for the coloring of several marine life forms, play a vital role in assessing the quality of seafood such as shrimp, lobsters, crabs, salmon, and tuna [4]. Marine bacteria generate a wide range of colors, like carotene, phenazine, violacein, melanin, pyrrole, and quinones [5]. Due to their critical involvement in numerous physiological processes, carotenoids are essential parts of living cells in almost all animals [1]. Algae and microalgae have been stated in scientific literature as excellent sources of bioactive substances (carotenoids) that may be used in the manufacturing of functional meals. Substances derived from marine animals are especially attractive due to the dynamic nature of the maritime environment, which involves varying quantities of salts. Fucoxanthin, the primary carotenoid found in brown seaweed, is the dominant marine carotenoid, comprising approximately 10% of all naturally existing carotenoids. Also, the production of peridinin from dinoflagellates and astaxanthin and canthaxanthin from algae is also notable and is increasing. Other carotenoids, such as halocynthiaxanthin, violaxanthin, tunaxanthin, and echinenone, have been found in substantial amounts in marine sources [6].

**Figure 1:**
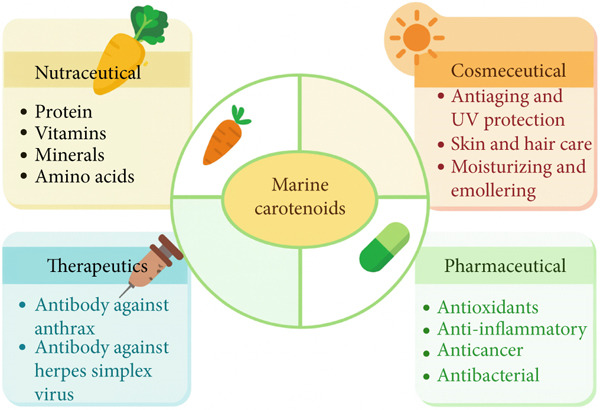
Flow diagram showing diverse applications of marine carotenoids.

Many studies have shown the biologically substantiated effects in cellular, animal, and human studies of natural substances derived from marine environments, such as carotenoids [7]. Carotenoids are a precursor of vitamin A, which is most notably shown by *β*‐cryptoxanthin and *β*‐carotene. Zeaxanthin and lutein help to absorb potentially harmful blue and near‐ultraviolet light to shield the macula lutea from harm caused by light‐related causes [8]. Carotenoids have also demonstrated other biological behaviors, including anti‐inflammatory, anticancer, antiobesity, antidiabetic, and cardiovascular protections [9]. Additionally, carotenoids possess potent antioxidant activity due to their capacity to eliminate free radicals and neutralize singlet oxygen. They protect against oxidative stress and prevent cell damage. Moreover, marine bacterial pigments possess many biological qualities, including antibacterial, antioxidant, and anticarcinogenic traits, and could be found in the food and pharmaceutical sectors. Hence, carotenoids are broadly used in the pharmaceutical, nutraceutical, and cosmetic sectors due to their multifunctional properties and several beneficial impacts on human health [5]. Furthermore, carotenoids have also been connected with other bioactivities, like positive effects on inflammation and cardiovascular disease, as well as possessing anticancer, antiobesity, and antidiabetic qualities [10], as seen in Figure 1. This review was aimed at examining the existing literature on the biological properties of marine carotenoids and their significant ability to improve human health.

## 2. Variety Among Aquatic Life Producing Carotenoids

The abundance of marine species that produce carotenoids is extensive and encompasses a broad spectrum of taxonomic groups. Marine algae, including both microscopic (microalgae) and macroalgae, are very productive in synthesizing a broad range of carotenoids. These forms of life involve dinoflagellates, diatoms, green algae, brown algae, and red algae. Carotenoids are generated by various marine bacteria and archaea, such as cyanobacteria, proteobacteria, and extremophilic archaea, which are present in deep‐sea and polar habitats [11]. Marine fungi and fungus‐like protists, namely, thraustochytrids, have the ability to create economically useful carotenoids such as astaxanthin and docosahexaenoic acid (DHA). Certain marine organisms, namely, crustaceans such as shrimp and crabs, can gather carotenoids from their food and use them for coloring and other physiological activities [12].

Carotenoids synthesized by these many marine creatures have a broad spectrum of structural variability, including notable substances such as fucoxanthin, astaxanthin, peridinin, and *β*‐carotene, which have been extensively investigated and hold significant economic value. Carotenoids have vital functions in the marine ecosystem and serve as essential components for the organisms that produce them. These functions include capturing light and protecting against damage in photosynthetic organisms, protecting against oxidative stress through antioxidant mechanisms, contributing to pigmentation and coloration, facilitating signaling and communication, and regulating growth and development [13].

## 3. Factors That Affect Carotenoid Levels in Marine Organisms

Carotenoid concentration in marine species is affected by a variety of variables, including environmental and biological influences. Gaining a complete understanding of these characteristics is essential to maximize the efficiency of producing and using marine carotenoids. Carotenoid production and accumulation are highly influenced by environmental variables. For example, the synthesis of carotenoids in photosynthetic organisms, such as microalgae and macroalgae, is significantly affected by the intensity of light and the wavelength of light. Increased light intensity can promote the synthesis of carotenoids as a strategy to defend against light‐induced damage, while certain wavelengths of light, such as blue and red, can selectively control the pathways involved in the formation of carotenoids [14]. Temperature is also a critical factor since various marine organisms have variable ideal temperature requirements. Organisms can undergo physiological stress due to extreme temperatures, which can trigger an increase in carotenoid production as a protective response. The presence of vital nutrients, like phosphorus, nitrogen, and trace elements, might affect the accumulation of carotenoids. When there is a lack of nutrients, it can cause an excessive synthesis of carotenoids as a reaction to stress. Salinity fluctuations may affect carotenoid synthesis in marine animals, namely, microalgae and halophilic bacteria. Higher levels of salinity can stimulate the production of some carotenoids, such as astaxanthin, in certain species [15]. Furthermore, biological variables have a part in the variation of carotenoid levels seen in marine animals. Marine species, including different strains within the same species, may have diverse carotenoid profiles and degrees of accumulation, which can be attributed to genetic and metabolic variations. The carotenoid concentration of marine creatures may vary throughout distinct growth phases and life stages. In some species, carotenoid levels tend to increase during the stationary phase or reproductive stages. The level of carotenoids in marine creatures may be affected by the expression and function of carotenogenic enzymes, as well as the control of carotenoid biosynthetic pathways. Various variables, including gene expression, enzyme kinetics, and feedback mechanisms, can regulate the synthesis of carotenoids [16].

## 4. Extraction Techniques of Marine Carotenoids

Carotenoids can be separated from various marine sources using a range of traditional and innovative extraction approaches, as shown in Table 1. Conventional approaches use different organic solvents such as acetone, methanol, and ethyl acetate [39]. Novel extraction technologies include enzyme‐assisted extraction, supercritical fluid extraction (SFE), microwave‐assisted extraction (MAE), ultrasound‐assisted extraction (UAE), high‐pressure homogenization (HPH), pulsed electric field (PEF), and moderate electric field (MEF) [17].

**Table 1:** A comparison of marine carotenoids by producing organisms, yield, extraction methods, and biological activity.

Types	Producing organisms	Yield	Extraction methods	Biological activity	References
Astaxanthin	Haematococcus pluvialis	19.1 mg/g DW	Pulsed electric field treatments	Antioxidant, immune system stimulation, antimicrobial cardiovascular protective, antiobesity, antiproliferative	[7, 17, 18]
	Geitlerinema amphibium	2.74 mg/g DW	Ultrasound (hexane, ethanol, acetone)		
	Chlorella vulgaris	70 *μ*g/g DW	Accelerated solvent extraction		
*α*‐Carotene	Chlorella vulgaris	50 *μ*g/g	Accelerated solvent extraction	Vision, provitamin A	[19, 20]
*α*‐Carotene, *β*‐carotene	Pyropia yezoensis	0.7 and 1.8 mg/g	Solvent extraction (methanol)	Vision, provitamin A	[19, 21]
*β*‐Carotene	Undaria pinnatifida	20.22 mg/g	SC‐CO_2_ extraction with ethanol	Vision, provitamin A, brain–cognitive functions, skin–UV protection, fertility, immune modulation/stimulation	[19, 22–24]
	Chlorella	1.4 mg/g *β*‐carotene	Solvent extraction (methanol sonication)		
	N. oleoabundans	17.4 mg/g extract	Pressurized liquid extraction		
Canthaxanthin	Chlorella vulgaris	32 *μ*g/g DW	Accelerated solvent extraction	Antioxidant, antibacterial immune system stimulation, cardiovascular	[18, 25, 26]
Fucoxanthin	Isochrysis zhangjiangensis	23 *μ*g/g DW	Solvent extraction (methanol)	Antioxidant, antibacterial, anticancer, antiobesity, neuroprotective, anti‐inflammatory, antiangiogenic, photoprotective, prevent osteoporosis, antiproliferative	[1, 23, 24, 27–29]
	Bryopsis sp.	3.44 ± 0.11 * μ*g/g DW	Solvent extraction (ice‐cold acetone)		
	Ceramium sp.	4.85 ± 0.04 * μ*g/g DW	Solvent extraction (ice‐cold acetone)		
	Gracilaria corticata	6.06 ± 0.05 * μ*g/g DW	Solvent extraction (ice‐cold acetone)		
	Grateloupia filicina	3.45 ± 0.06 * μ*g/g DW	Solvent extraction (ice‐cold acetone)		
	Sargassum wightii	3.13 ± 0.09 * μ*g/g DW	Solvent extraction (ice‐cold acetone)		
	Ulva prolifera	0.69 ± 0.09 * μ*g/g DW	Solvent extraction (ice‐cold acetone)		
	Sargassum horneri	0.77 mg/g DW	SC‐CO_2_ extraction with ethanol		
	Alaria esculenta	0.82 mg/g DW	Enzymatic extraction		
	Padina tetrastromatica	0.75 mg/g DW	Ultrasound extraction		
	Alaria esculenta	73.0 ± 14.0 mg (100 g^−1^ FD)	Ultrasound extraction		
Halocynthiaxanthin	Halocynthia roretzi			Antiproliferative and apoptosis, anticancer	[30]
Lycopene	Blakeslea trispora			Skin–UV protection, heart health, cancer prevention, anti‐inflammatory, antimicrobial, anti‐inflammatory, anticancer, and antioxidant activity	[31, 32]
Lutein	Desmodesmus sp.	5.11 mg/g DW	Solvent extraction (methanol sonication)	Anti‐inflammatory, ocular‐protective, antibacterial, antioxidative, neuroprotective, cardioprotective, antiplasmodial	[1, 20, 21, 23, 33]
	Chlorella	3.22 mg/g DW	Solvent extraction (methanol sonication)		
	Chlorococcum sp.	15.5 mg/g DW	Solvent extraction (methanol)		
	Scenedesmus sp.	10.7 mg/g DW	Solvent extraction (methanol)		
	N. oleoabundans	62.6 mg/g extract	Pressurized liquid extraction		
	Geitlerinema amphibium	5.49 mg/g DW	Ultrasound‐aided solvent extraction (hexane, ethanol, acetone)		
	Gloeothece sp.	2.9 mg/g DW	Pressurized solvent extraction		
	Bryopsis sp.	4.06 ± 0.06 * μ*g/g DW	Solvent extraction (ice‐cold acetone)		
	Ceramium sp.	3.26 ± 0.07 * μ*g/g DW	Solvent extraction (ice‐cold acetone)		
	Chaetomorpha antennina	141.30 ± 0.18 * μ*g/g DW	Solvent extraction (ice‐cold acetone)		
	Cladophora sp.	248.67 ± 0.13 * μ*g/g DW	Solvent extraction (ice‐cold acetone)		
	Gracilaria corticata	0.26 ± 0.05 * μ*g/g DW	Solvent extraction (ice‐cold acetone)		
	Grateloupia sp.	166.58 ± 0.42 * μ*g/g DW	Solvent extraction (ice‐cold acetone)		
	Grateloupia filicina	18.38 ± 0.23 * μ*g/g DW	Solvent extraction (ice‐cold acetone)		
	Sargassum wightii	0.46 ± 0.05 * μ*g/g DW	Solvent extraction (ice‐cold acetone)		
	Ulva compressa	4.68 ± 0.51 * μ*g/g DW	Solvent extraction (ice‐cold acetone)		
	Ulva fasciata	0.90 ± 0.12 * μ*g/g DW	Solvent extraction (ice‐cold acetone)		
	Ulva lactuca	21.13 ± 0.07 * μ*g/g DW	Solvent extraction (ice‐cold acetone)		
	Ulva prolifera	10.23 ± 0.12 * μ*g/g DW	Solvent extraction (ice‐cold acetone)		
	Chlorella vulgaris	0.753 mg/g DW	Pulsed electric field (96% ethanol)		
	Pyropia yezoensis	1.4 mg/g DW	Solvent extraction (methanol)		
	Chlorella sorokiniana	20.69 ± 1.2 mg/g DW	Microwave‐assisted alkali (8.16 M KOH)		
	Chlorella vulgaris	90 *μ*g/g DW	Accelerated solvent extraction		
Neoxanthin	Chlamydomonas reinhardtii			Antioxidant	[34]
Peridinin	Heterocapsa triquetra			Antioxidant, antiproliferative, anticancer, anti‐inflammatory	[35]
Spirilloxanthin	Rhodoplanes roseus			Antioxidant	[36]
Zeaxanthin	Desmodesmus sp.	0.28 mg/g zeaxanthin DW	Solvent extraction (methanol sonication)	Eye health, antimalarial activity	[1, 21–24, 37, 38]
	N. oleoabundans	6 mg/g extract	Pressurized liquid extraction		
	Cyanobacterium aponinum	3.17 mg/g DW	Solvent extraction (acetone 100%)		
	Bryopsis sp.	1.62 ± 0.03 * μ*g/g DW	Solvent extraction (ice‐cold acetone)		
	Ceramium sp.	0.66 ± 0.05 * μ*g/g DW	Solvent extraction (ice‐cold acetone)		
	Chaetomorpha antennina	34.58 ± 0.41 * μ*g/g DW	Solvent extraction (ice‐cold acetone)		
	Cladophora sp.	50.20 ± 0.10 * μ*g/g DW	Solvent extraction (ice‐cold acetone)		
	Gracilaria corticata	0.65 ± 0.04 * μ*g/g DW	Solvent extraction (ice‐cold acetone)		
	Grateloupia sp.	36.34 ± 0.21 * μ*g/g DW	Solvent extraction (ice‐cold acetone)		
	Grateloupia filicina	2.16 ± 0.03 * μ*g/g DW	Solvent extraction (ice‐cold acetone)		
	Ulva compressa	3.95 ± 0.18 * μ*g/g DW	Solvent extraction (ice‐cold acetone)		
	Ulva fasciata	0.25 ± 0.04 * μ*g/g DW	Solvent extraction (ice‐cold acetone)		
	Pyropia yezoensis	0.15 mg/g	Solvent extraction (methanol)		
	Ulva lactuca	11.26 ± 0.12 * μ*g/g DW	Solvent extraction (ice‐cold acetone)		
	Ulva prolifera	9.47 ± 0.07 * μ*g/g DW	Solvent extraction (ice‐cold acetone)		

## 5. Chemical Composition of Carotenoids

**Figure 2:**
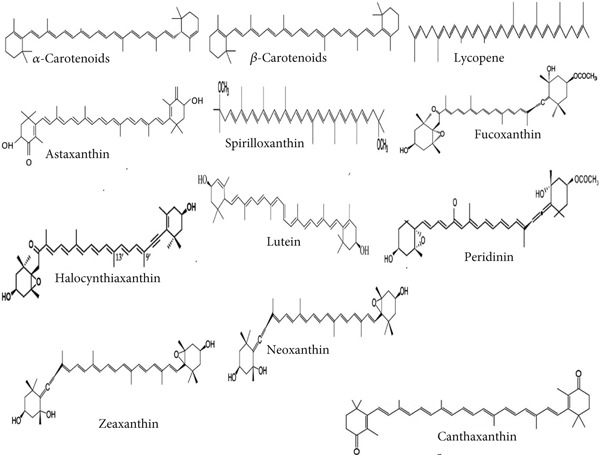
The chemical formula of the main marine carotenoids.

Carotenoids are a group of highly potent natural dyes made up of eight parts, each consisting of five carbons with changing single and double bonds. The carotenoid chain may bond with an oxygen functional group (astaxanthin) or a cyclic group (*α* and *β*‐carotene) based on the type of metabolite [40]. There are more than 700 types of carotenoids, of which lutein, astaxanthin, *α*‐ and *β*‐carotene, zeaxanthin, etc. have been used in industrial manufacturing [41, 42]. Carotenoids are produced by the connection of two molecules of C_20_ geranyl diphosphate. Carotenoids are characterized by the presence of a polyisoprenoid framework, which is a long chain of double bonds that are conjugated, meaning they are connected in a continuous manner. Furthermore, carotenoids have a nearly symmetrical configuration around the double bond of the core [43]. Carotenoids can be classified into provitamin A (e.g., *α*‐ and *β*‐carotene and *β*‐cryptoxanthin) and nonprovitamin A compounds [44]. Carotenoids can be grouped according to their functional classes as follows: The two types of pigments found in plants are xanthophylls, like zeaxanthin and lutein, which include oxygen as a functional group, and carotenes, such as *α*‐ and *β*‐carotene and lycopene, which merely have a hydrocarbon chain without any functional group [43]. Apocarotenoids, which include retinoids, vitamin A, *β*‐ionone, and *α*‐ionone aromatic volatile chemicals, are produced from carotenoids by oxidative cleavage with the help of carotenoid cleavage dioxygenases [45]. Secondary carotenoids are classified according to their distinct functional groups, which include the hydroxy group (zeaxanthin and lutein), keto (canthaxanthin and astaxanthin), methoxy (spirilloxanthin), and epoxy (violaxanthin, neoxanthin, and fucoxanthin), as shown in Figure 2. The framework of secondary carotenoids includes hydrogen in addition to carbon. Green algae also create all the xanthophylls that are synthesized by higher plants [46]. Carotenoids are typically hydrophobic substances that are slightly soluble in water and function inside the hydrophobic regions of the cell. The presence of functional polar groups on the polyene chain could alter the polarity of carotenoids, potentially affecting their distribution inside biomembranes and their interactions with other molecules [47].

## 6. Biosynthesis of Carotenoids

Although biosynthesized carotenoids vary between species, almost every species of photosynthetic plant or microalgae has a typical main metabolic pathway, as seen in Figure 3a,b. The identical C_5_ construction block, isopentenyl pyrophosphate, or its isomer, dimethylallyl diphosphate, is the starting point for all pathways. It is generated from pyruvate and glyceraldehyde 3‐phosphate (the plastidic methylerythritol 4‐phosphate pathway) or acetyl‐CoA (the cytosolic mevalonic acid pathway method). The methylerythritol 4‐phosphate pathway was proposed to produce isopentenyl pyrophosphate or dimethylallyl diphosphate, which are used in the synthesis of carotenoids even though both pathways result in the same product [48]. Next, in an abundance of enzymes, the intermediate C_15_ farnesyl diphosphate or C_20_ geranylgeranyl diphosphate is created through sequential chain lengthening in the head‐to‐tail fashion. Head‐to‐head condensation occurs after this stage and yields phytoene, the C_40_ carotenoid. Algae and higher plants can produce *β*‐carotene when desaturase is present (bacteria and fungi have slightly different metabolic processes). After that, lycopene, the first red‐colored carotenoid, forms [49]. Furthermore, through two different cyclization processes, the widely known *α*‐ or *β*‐carotene structures are generated. The extremely diversified carotenoid family can then result from further chain changes such as hydroxylation, epoxidation, ketolation, glycosylation, and oxygen cleavage [48]. However, astaxanthin is not present in many higher plants; instead, photosynthetic microalgae typically synthesize it from canthaxanthin or zeaxanthin [50]. The cytoplasm is where some processes in the manufacture of carotenoids are found in the chloroplast. Because it completes a rate‐limiting step, phytoene synthase is one of the essential enzymes for carotenoid production in photosynthetic organisms. Stress in the environment can elevate the expression of phytoene synthase or other synthase genes. Numerous reviews on enzymes involved in the carotenoids′ production pathway are available [51].

Figure 3(a, b) Overview of carotenoid biosynthesis. From MEP/MVA precursors to GGPP, then phytoene → lycopene → *α*/*β*‐carotene, branching to major xanthophylls (lutein, zeaxanthin, canthaxanthin, and astaxanthin).

**Figure:**
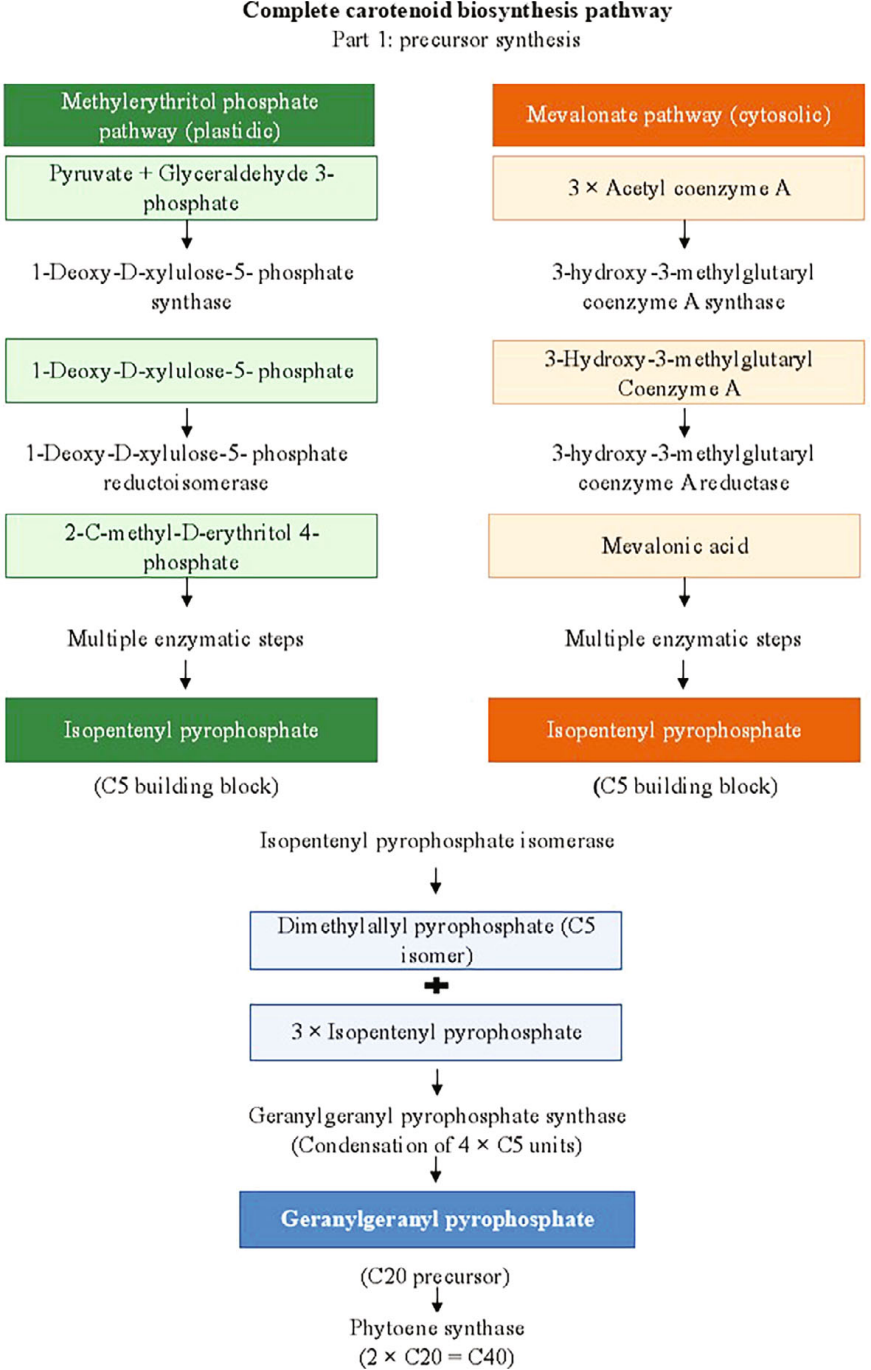
(a)

**Figure:**
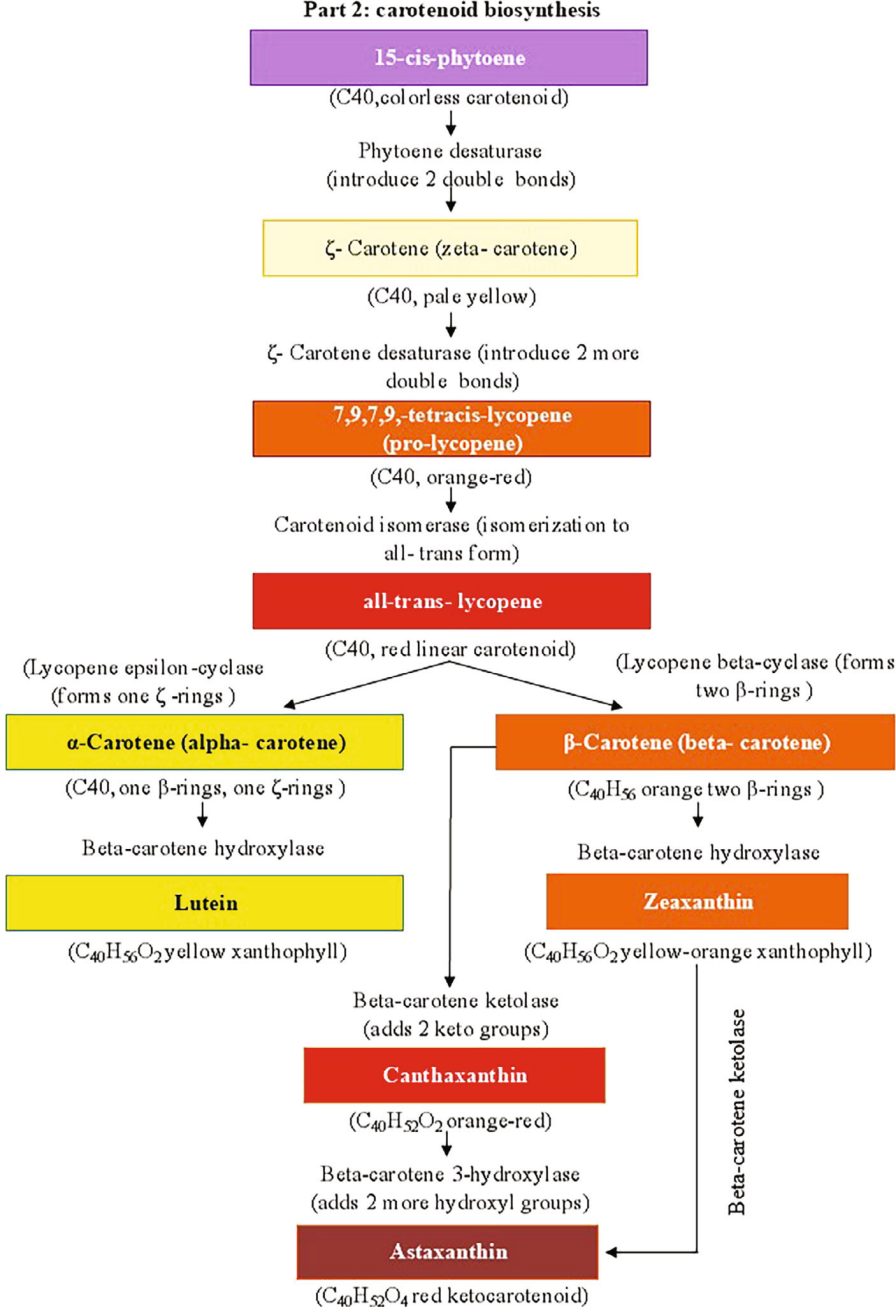
(b)

## 7. Types, Mechanisms of Action, Bioavailability, and Efficacy of Carotenoids

### 7.1. Alpha‐ and Beta‐Carotene (*α*‐ and *β*‐Carotene)

*α*‐ and *β*‐carotene are types of carotenoids composed only of carbon and hydrogen. Both carotenes are the most abundant carotenes manufactured by algae strains such as *Dunaliella salina*, *Haematococcus* sp., *Chlamydocapsa* sp., and *Chlorococcum* sp. (Chlorophyta). *β*‐Carotene, a provitamin A, is especially important for immune system health and normal vision [52]. Its high demand extends to its use in multivitamin preparations and as a culinary coloring agent in a wide range of foods and beverages. Beyond its nutritional value, *β*‐carotene has been shown to reduce the risk of age‐related macular degeneration (AMD) and is utilized in animal feed. Furthermore, it has been explored in the treatment of conditions such as asthma and cardiovascular disease [53] as shown in Table 1.

The ways carotenoids work their magic are complex, involving various biochemical pathways. They are powerful antioxidants because their conjugated double‐bond system allows them to efficiently quench singlet oxygen and scavenge other free radicals [53]. This helps protect cells from oxidative damage, a key factor in aging, chronic diseases, and inflammation. Their unique electron delocalization helps stabilize free radicals, preventing further damage from lipid peroxidation and DNA damage. Carotenoids can also influence gene expression and cell signaling. For example, *β*‐carotene has been shown to modulate the transcription of various cytokines. Katsuura et al. [54] found that *β*‐carotene supplementation repressed the transcription of interleukin‐1*β* (IL‐1*β*), interleukin‐6 (IL‐6), and IL‐12 p40, suggesting an anti‐inflammatory role by reducing the production of proinflammatory cytokines.

Their impact on the immune system is significant. Studies show a complex, dose‐dependent effect of *β*‐carotene on human NK cells in vitro; lower doses reduced the tumorolytic effect, while higher concentrations significantly increased it [55]. This suggests there might be an optimal range for its immune benefits. *β*‐Carotene has also been shown to increase lymphoid cells with markers for NK cells, IL‐2, and transferrin receptors in people taking oral supplements [56]. Another study found that *β*‐carotene promotes thymus gland growth and an increased count of thymic small lymphocytes [57], pointing to its role in T cell development.

**Figure 4:**
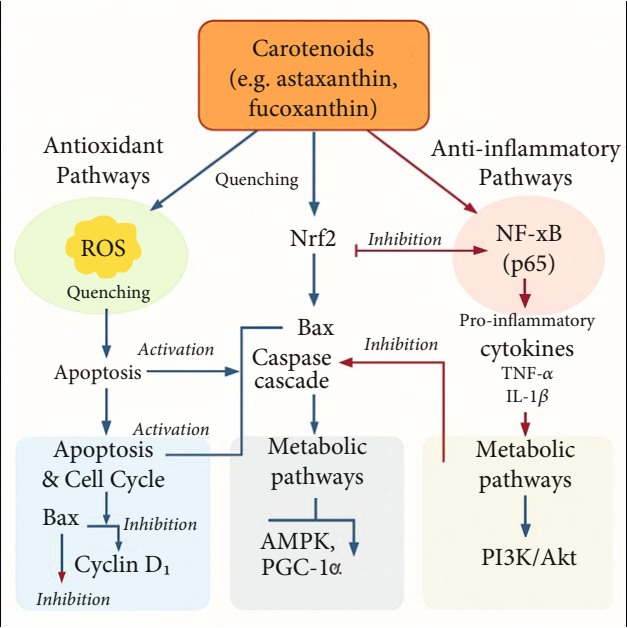
Schematic diagram illustrates the major cellular signaling pathways modulated by marine carotenoids such as astaxanthin, fucoxanthin, peridinin, and lycopene. These compounds exert multifaceted effects beyond antioxidant activity, including the following: Antioxidant pathways: Carotenoids scavenge reactive oxygen species (ROS) and activate the Nrf2 pathway, leading to the transcription of antioxidant response element (ARE)–driven genes, such as *HO-1*, *NQO1*, *SOD*, and *catalase*. Anti‐inflammatory pathways: They inhibit the nuclear translocation of NF‐*κ*B (p65) by stabilizing I*κ*B, which downregulates proinflammatory cytokines (*TNF-α*, *IL-1β*). Apoptosis and cell cycle regulation: Carotenoids induce apoptosis via Bax‐mediated activation of caspase cascades (caspase‐8, caspase‐9, caspase‐3), decrease Bcl‐2 expression, and inhibit cell proliferation through downregulation of cyclin D1. Metabolic pathways: Modulation of PI3K/Akt and AMPK/PGC‐1*α* signaling has been reported, enhancing mitochondrial biogenesis and improving insulin sensitivity in some models.

Oral administration of *β*‐carotene specifically enhanced Peyer′s patch (PP) cell production of IL‐2 while leaving IL‐4 levels unchanged. When combined with capsaicin, *β*‐carotene led to significantly higher levels of IFN‐*γ* and IL‐5 [58]. These findings indicate that *β*‐carotene can selectively modulate Th cytokine production, shifting the immune response toward a Th1‐type profile, crucial for fighting viruses and tumors as shown in Figure 4.

Recent research has further elucidated that *β*‐carotene′s impact on IgA production is mediated through the activation of specific dendritic cell populations in the gut, which in turn promote the differentiation of B cells into IgA‐secreting plasma cells, thus reinforcing its role in mucosal immunity [59]. *β*‐Carotene may also have anti‐inflammatory properties against DNA viruses, especially human herpes simplex virus, by blocking cytokine expression in Suid herpesvirus‐induced inflammation through the inactivation of NF‐*κ*B [60], as shown in Table 2.

**Table 2:** Evidence summary for *α*‐ and *β*‐carotene health outcomes.

Outcome	In vitro evidence	Animal studies	Human studies	Strength of evidence and translational relevance
Antioxidant activity	High ROS quenching via conjugated double‐bond system; efficiently quench singlet oxygen and scavenge free radicals; electron delocalization stabilizes free radicals [53]	Significant reduction in lipid peroxidation in hepatic tissues; synthesis of glutathione and *β*‐carotene accumulation in murine macrophages directly related to cellular lipid peroxidation [55]	Increased plasma antioxidant capacity and serum levels [53]	Strong (+++)—Well‐established antioxidant effects across all models; clinically relevant via dietary intake
Immune modulation	Complex dose‐dependent effect on human NK cells; lower doses reduced tumorolytic effect, higher concentrations significantly increased it [55]; modulates transcription of cytokines [54]	Enhanced thymus gland growth and increased count of thymic small lymphocytes [57]; *β*‐carotene specifically enhanced Peyer′s patch cell production of IL‐2; combined with capsaicin led to higher IFN‐*γ* and IL‐5 levels [58]; IgA production mediated through dendritic cell activation [59]	*β*‐Carotene increased lymphoid cells with markers for NK cells, IL‐2, and transferrin receptors in oral supplement users [56]; supplementation repressed transcription of IL‐1*β*, IL‐6, and IL‐12 p40 [54]	Strong (+++)—Consistent immunomodulatory actions; promising translational relevance, especially in age‐related immune decline
Cancer prevention	*α*‐Carotene more potently inhibited GOTO human neuroblastoma cell proliferation than *β*‐carotene and halted cell cycle at G0/G1 phase [61]; may more effectively induce apoptosis and inhibit angiogenesis [62]	*α*‐Carotene demonstrated greater potency than *β*‐carotene in suppressing skin and lung carcinogenesis and decreasing hepatomas in mice with spontaneous liver carcinogenesis [61, 62]	Mixed epidemiological data; supplementation studies show complex results	Mixed (+/−)—Strong preclinical evidence; translation limited due to population‐specific risk profiles
Cardiovascular health	General antioxidant and anti‐inflammatory properties [53]	Animal studies on lipid markers and atherosclerotic lesions (referenced but not detailed in provided text)	Higher blood levels of *α*‐carotene inversely associated with cardiovascular disease mortality [63]	Moderate (++)—Positive dietary associations; supplements show no additional benefit; more targeted trials needed
Cognitive function	Neuroprotection in neuronal cultures (limited data in provided text)	Preservation of motor function and reduced neuroinflammation in aging models (referenced but not detailed)	Positive association between higher circulating *α*‐carotene levels and better cognitive function, particularly global cognition and semantic memory, in older adults [64]	Moderate (++)—Encouraging early findings; clinical significance under investigation
Muscle strength	—	Improved muscle integrity and function in aged rodents (referenced but not detailed)	Serum *α*‐carotene levels positively associated with muscle strength in older adults, distinguishing it from other antioxidants like vitamin E and *β*‐carotene; unique role in maintaining muscle integrity during aging [65]	Moderate (++)—Emerging evidence for *α*‐carotene′s role in sarcopenia prevention; mechanistic pathways need clarification

*Note:* (+++) strong consistent evidence, (++) moderate evidence, (+) limited evidence, (+/−) mixed/inconclusive evidence.

NF‐*κ*B is a crucial transcription factor in inflammation, so its inhibition can suppress the production of proinflammatory mediators. Interestingly, some studies suggest that *β*‐carotene can act as a pro‐oxidant under certain conditions, especially at high concentrations or with specific cofactors. The synthesis of glutathione (an endogenous antioxidant) and the accumulation of *β*‐carotene in murine macrophage cells were directly related to cellular lipid peroxidation, influenced by both exposure duration and dose [55]. This complex behavior shows how important context and concentration are in determining *β*‐carotene′s overall effect.

While *β*‐carotene often takes center stage due to its higher provitamin A activity and abundance, *α*‐carotene is also a significant carotenoid with unique contributions to human health. Like *β*‐carotene, it is a hydrocarbon carotenoid, meaning it consists solely of carbon and hydrogen. However, its structure differs slightly from *β*‐carotene: *α*‐carotene possesses a *β*‐ionone ring at one end and an *α*‐ionone ring (also known as an *ϵ*‐ring) at the opposite end, whereas *β*‐carotene has two *β*‐ionone rings. This subtle structural difference impacts its biological activity [66]. *α*‐Carotene shares many of the general mechanisms of action attributed to carotenoids, particularly its strong antioxidant properties. Its conjugated double‐bond system enables it to effectively quench singlet oxygen and scavenge free radicals, thereby protecting cells from oxidative damage that contributes to aging and disease [65].

Beyond its direct antioxidant role, *α*‐carotene exhibits specific effects on cellular processes:

• Anticarcinogenic activity: Emerging research suggests *α*‐carotene may possess even greater anticarcinogenic activity than *β*‐carotene in certain contexts. It has been shown to inhibit the proliferation of GOTO human neuroblastoma cells more potently than *β*‐carotene and can halt the cell cycle at the G0/G1 phase [61]. In mouse models, *α*‐carotene has demonstrated greater potency than *β*‐carotene in suppressing skin and lung carcinogenesis and decreasing the number of hepatomas in mice with spontaneous liver carcinogenesis. The exact biochemical pathways for this enhanced anticancer effect are still being investigated, but it may involve differential modulation of cell growth and signaling pathways. *α*‐Carotene may exert its superior anticancer effects by more effectively inducing apoptosis and inhibiting angiogenesis in specific cancer cell lines compared to *β*‐carotene, possibly through distinct interactions with intracellular signaling molecules [62].
• Cardiovascular health: Like *β*‐carotene, *α*‐carotene contributes to cardiovascular health by acting as an antioxidant and reducing inflammation. Higher blood levels of *α*‐carotene have been inversely associated with cardiovascular disease mortality [63].
• Cognitive function: Newer research suggests a positive association between higher circulating *α*‐carotene levels and better cognitive function, particularly global cognition and semantic memory, in older adults. This protective effect is likely linked to its antioxidant capacity and ability to mitigate oxidative stress in brain tissue [64].
• Muscle strength: Recent findings also indicate that serum *α*‐carotene levels are positively associated with muscle strength in older adults, distinguishing it from other antioxidants like vitamin E and *β*‐carotene in some studies. This suggests a unique role for *α*‐carotene in maintaining muscle integrity and function during aging, potentially by reducing oxidative stress within muscle cells [65].

The effectiveness and absorption of different carotenoids vary due to their distinct chemical structures, absorption mechanisms, and metabolic pathways. While *α*‐ and *β*‐carotene are both carotenes, their subtle structural differences can affect how they interact with cell membranes and how efficiently they are absorbed. While both *α*‐ and *β*‐carotene are provitamin A carotenoids, their efficiency in conversion to vitamin A differs significantly. *β*‐Carotene, with its two *β*‐ionone rings, can theoretically be cleaved to yield two molecules of biologically active retinol (vitamin A). In contrast, *α*‐carotene, having only one *β*‐ionone ring, is converted to just one molecule of biologically active retinol after central cleavage, along with one molecule of *α*‐retinol, which has negligible vitamin A activity. Consequently, *α*‐carotene has less vitamin A activity than *β*‐carotene [67]. Despite its lower provitamin A activity, studies have shown complex patterns regarding *α*‐carotene′s bioavailability and efficacy compared to *β*‐carotene:

• Apparent bioavailability: Some research suggests that when consuming comparable amounts of *α*‐carotene‐ and *β*‐carotene‐rich foods, *α*‐carotene may result in higher blood concentrations compared to *β*‐carotene. This implies that *α*‐carotene from its primary food sources might have greater apparent bioavailability than *β*‐carotene, though further research is needed to fully understand the underlying factors [68].
• Food matrix and processing: The bioavailability of both *α*‐ and *β*‐carotene is significantly influenced by the food matrix and processing methods. For example, absorption of both carotenes was approximately twofold greater from carrot puree than from boiled–mashed carrots. Processing techniques that disrupt plant cell walls and release carotenoids generally enhance their absorption [67].
• Interindividual variability: There is considerable interindividual variability in the conversion efficiency of provitamin A carotenoids, including *α*‐carotene, to vitamin A. This variability highlights that dietary intake alone may not guarantee sufficient levels or conversion in all individuals [69].

The effects of *β*‐carotene and related carotenoids, such as canthaxanthin and astaxanthin, were examined on the growth and capacity of murine immunocompetent cells via the in vitro cell cultures. The findings demonstrated that *β*‐carotene, canthaxanthin, and astaxanthin all have strong, but distinct, stimulatory effects on the growth of BALB/c mice′s spleen and thymocyte cells. At specific doses, the release of IL‐1 and tumor necrosis factor‐*α* (TNF‐*α*) from mouse peritoneal adherent cells was markedly enhanced by the three carotenoids. However, astaxanthin exhibited more cytokine‐inducing activities compared to canthaxanthin and *β*‐carotene, respectively [70]. The studies were the first to show that the carotenoid extract of *Dunaliella salina*, which contains lutein, zeaxanthin, *α*‐carotene, and *β*‐carotene, can inhibit the expression of inducible nitric oxide synthase (iNOS) and cyclooxygenase‐2 (COX‐2) in LPS‐activated RAW cells. Furthermore, it can also reduce the production of NO, PGE2, and proinflammatory cytokines (TNF‐*α*, IL‐1*β*, and IL‐6) [71]. The extract showed anti‐inflammatory properties by inhibiting NF‐*κ*B activation and JNK phosphorylation [72]. Like zeaxanthin supplements, meso‐zeaxanthin is a xanthophyll carotenoid with strong antioxidant activity that also reduces inflammation in the BALB/c mouse model [73]. Subsequent examination has shown that lutein and *β*‐carotene, while to a lesser extent than ASTA, improve the generation of antibodies against T‐dependent antigens in elderly B6 mice, but not against T‐independent antigens. Table 2 summarizes the evidence for *α*‐ and *β*‐carotene across in vitro, animal, and human studies, highlighting the progression from mechanistic understanding to clinical validation.

### 7.2. Lycopene

Lycopene is a type of carotenoid composed of hydrocarbons only. Lycopene production by a fungus (*Blakeslea trispora*) has anti‐inflammatory activity by reducing proinflammatory reactions that are triggered by lipopolysaccharide (LPS) by enhancing the integrity of the vascular barrier, inhibiting barrier permeability and the production of cell adhesion molecules (CAMs), and preventing leukocyte adherence and transendothelial migration. Lycopene′s anti‐inflammatory effects are mediated by TNF‐*α* production and NF‐*κ*B expression [74]. Moreover, lycopene reduced the levels of intercellular adhesion molecule‐1 (ICAM‐1) generated by TNF‐*α* in human umbilical vein endothelial cells (HUVECs) but had no impact on the expression of COX‐2 or platelet endothelial cell (EC) adhesion molecules. The anti‐inflammatory qualities of lycopene may potentially be used to prevent cardiovascular disease. This is due to its ability to decrease TNF‐*α*‐induced I*κ*B phosphorylation, NF‐*κ*B expression, and translocation of NF‐*κ*B p65 from the cytosol to the nucleus. Additionally, lycopene had little impact on ICAM‐1 expression caused by IFN‐*γ*, indicating that lycopene mainly influences the signaling pathway induced by TNF‐*α* [75].

At the molecular level, lycopene′s anti‐inflammatory activity involves multiple interconnected signaling pathways. The carotenoid functions as a potent singlet oxygen quencher with a rate constant of 1.6 × 10^10^ *M* ^−1^ *s* ^−1^, which is approximately twice that of *β*‐carotene [76]. Lycopene′s antioxidant mechanism involves the delocalization of unpaired electrons across its extended conjugated double bond system, consisting of 11 conjugated double bonds, making it one of the most effective biological antioxidants [77]. The compound directly scavenges reactive oxygen species (ROS), including superoxide anions, hydroxyl radicals, and nitrogen dioxide, thereby preventing oxidative stress–induced activation of redox‐sensitive transcription factors such as NF‐*κ*B and activator protein‐1 (AP‐1) [78].

The inhibition of NF‐*κ*B signaling by lycopene occurs through multiple mechanisms: (1) prevention of I*κ*B kinase (IKK) activation by blocking upstream kinases, including mitogen‐activated protein kinase kinase kinase‐1 (MEKK‐1) and NF‐*κ*B‐inducing kinase (NIK); (2) direct interaction with the RelA/p65 subunit, preventing its nuclear translocation; and (3) enhancement of I*κ*B*α* protein stability through inhibition of proteasomal degradation [79]. Furthermore, lycopene modulates the phosphatidylinositol 3‐kinase (PI3K)/Akt signaling pathway, leading to increased expression of nuclear factor erythroid 2‐related factor 2 (Nrf2) and subsequent upregulation of antioxidant response element (ARE)–driven genes including heme oxygenase‐1 (HO‐1), NAD(P) H quinone oxidoreductase 1 (NQO1), and glutathione S‐transferase [80, 81]. Recent studies have demonstrated that lycopene′s anti‐inflammatory effects are also mediated through epigenetic mechanisms. Lycopene treatment (10 *μ*M for 24 h) significantly reduces DNA methylation at the promoter regions of anti‐inflammatory genes, including IL‐10 and transforming growth factor‐*β* (TGF‐*β*), while increasing histone H3 acetylation at these loci. This epigenetic modulation is facilitated by lycopene′s ability to inhibit DNA methyltransferase (DNMT) activity by approximately 40% and enhance histone acetyltransferase (HAT) activity by 60% [82, 83]. Additionally, lycopene influences microRNA expression profiles, particularly upregulating miR‐146a and miR‐155, which are crucial negative regulators of inflammatory responses through targeting of interleukin‐1 receptor‐associated kinase 1 (IRAK1) and tumor necrosis factor receptor‐associated factor 6 (TRAF6) [84].

The bioavailability of lycopene varies significantly depending on its source, processing method, and coadministration with other nutrients. Lycopene from tomato paste exhibits 2.5‐fold higher bioavailability compared to fresh tomatoes due to thermal processing that converts the predominant trans‐isomer (> 95% in raw tomatoes) to more bioavailable cis‐isomers [85]. The presence of dietary fats enhances lycopene absorption by 3–5‐fold, as demonstrated in studies where lycopene bioavailability increased from 0.3% to 1.5% when consumed with olive oil [86]. Lycopene absorption follows first‐order kinetics with a peak plasma concentration (*C*_max_) of 0.5–1.0 *μ*M achieved 24–48 h postingestion and a biological half‐life of 2–3 days [87]. Gender differences in lycopene metabolism have been observed, with women showing higher plasma lycopene levels compared to men when consuming equivalent doses, potentially due to differences in lipoprotein metabolism and hormonal influences on carotenoid transport [88]. Genetic polymorphisms in key enzymes involved in lycopene metabolism significantly affect individual responses to supplementation. Variants in the *β*‐carotene oxygenase 1 (BCO1) gene, particularly the A379V polymorphism, result in 40%–60% reduced lycopene cleavage activity, leading to higher plasma lycopene levels but potentially reduced formation of bioactive metabolites [89]. Similarly, polymorphisms in the scavenger receptor class B type 1 (SCARB1) gene affect lycopene uptake efficiency, with the rs5888 variant associated with 25% reduced absorption. These genetic variations contribute to the wide interindividual variability (3–10‐fold) observed in plasma lycopene responses to standardized supplementation protocols [90, 91].

The pretreatment of human THP‐1 macrophages with lycopene led to a considerable reduction in the RNA and protein levels of IL‐8 production caused by the extract of cigarette smoke extract; NF‐*κ*B inactivation is the molecular mechanism that mediates this effect. According to Simone et al., NF‐*κ*B inactivation has been linked to both PPAR and redox signaling activation. At a dose of 5 *μ*M, lycopene has favorable effects on both NK cell viability and cytotoxicity. Its ability to prevent NK cells from undergoing apoptosis is linked to a decrease in the expression of the caspase 3 and 9 genes [92]. Furthermore, lycopene did not influence the expression of NKG2A, NKG2D, NKp30, and NKp44, among other functional receptors in NK cells. After 7 days, lycopene treatment boosted IFN‐*γ* expression at both gene and protein levels [93]. Lycopene has been shown to increase the synthesis of TNF‐*α* and IL‐1*β* in human PBMC in a dose‐dependent manner while decreasing the secretion of IL‐2, IL‐10, and IFN‐*γ*. However, IL‐6 and the IL‐1 receptor antagonist remained unaffected. Lycopene can exacerbate inflammatory responses, as evidenced by increased production of proinflammatory cytokines (TNF‐*α* and IL‐1*β*) and decreased secretion of anti‐inflammatory cytokines (IL‐10) [94]. However, several studies conducted in adipose tissues and adipocyte models have shown that lycopene effectively reduces the production of proinflammatory cytokines and chemokines, including IL‐6, IL‐1*β*, and MCP‐1, at both mRNA and protein levels. The presence of a high concentration of lycopene (10 *μ*mol·L^−1^) resulted in significant antiangiogenic effects. This could be attributed to the increased expression of IFN‐*γ* (~531%) and IL‐12 (~163%) in HUVEC [95].

Lycopene may be beneficial in preventing or treating acute pancreatitis by reducing intracellular levels of ROS in pancreatic acinar cells, thus limiting the activation of NF‐*κ*B and the production of inflammatory cytokines such as IL‐6 [96]. According to Martorchino et al., lycopene′s anti‐inflammatory properties in RAW 264.7 macrophages were linked to a reduction in LPS‐stimulated migration [97]. The study by Hadad and Levy found that preincubation of mouse peritoneal macrophages with lycopene at a concentration of approximately 1 *μ*M, lutein at a concentration of approximately 1 *μ*M, and *β*‐carotene at a concentration of approximately 2 *μ*M, before adding LPS, led to a synergistic suppression of NO, PGE2, and superoxide generation [98]. The decrease in iNOS, COX‐2, and NADPH oxidase expression at both the mRNA and protein levels, together with a combined suppression of TNF‐*α* production, was responsible for this effect. According to Rafi et al. [99], lycopene administration (~10 *μ*M) did reduce LPS‐induced iNOS protein and NO generation (~40%) in RAW 2647 dose‐dependently. However, studies have shown that the addition of lycopene to one′s diet could reduce general allergic inflammation, especially in the lungs, by decreasing the body′s reaction to Th2 cytokines. Lycopene inhibited the expression of the Th2 transcription factor GATA‐3, the cytokine IL‐4, the activity of eosinophil peroxidase and MMP‐9, and the infiltration of inflammatory leukocytes (including neutrophils, eosinophils, lymphocytes, and macrophages) into bronchoalveolar lavage fluid [100]. However, in a mouse model of postmyocardial infarction remodeling, lycopene has been shown to reduce inflammation and apoptosis (e.g., reduction of caspase‐3, caspase‐8, and caspase‐9 expression) by blocking the NF‐*κ*B signaling pathway (e.g., NF‐*κ*B p65 phosphorylation) [29]. Lycopene has been found to inhibit the release of high mobility group 1 (HMGB1) and proinflammatory signaling responses mediated by HMGB1 in both primary HUVEC and animals. This is achieved by downregulating the cell surface expression of CAMs, as well as HMGB1 receptors, TLR‐2 and TLR‐4, and receptors for advanced glycation end products, in response to LPS. In addition, it has been shown to trigger proinflammatory reactions in ECs [101]. Lycopene has been demonstrated to have xanthophylls, which comprise 40% lutein and 60% zeaxanthin, that control the expression of pro‐ and anti‐inflammatory cytokines in a variety of hen and chick tissues. In the liver, duodenum, and jejunum of hens, dietary xanthophyll decreases the expression of proinflammatory cytokines (e.g., IFN‐*γ*, IL‐6, IL‐1*β*, and LITAF). It enhances the expression of anti‐inflammatory cytokines (e.g., IL‐4 and IL‐10) in these organs [102]. Also, a systematic comparison of lycopene bioactivities across research models is presented in Table 3, enabling evaluation of the translational pathway from cellular mechanisms to clinical applications.

**Table 3:** Evidence summary for lycopene health outcomes.

Outcome	In vitro evidence	Animal studies	Human studies	Strength of evidence and translational relevance
Antioxidant activity	Potent ROS scavenging; singlet‐oxygen quenching kq ≈ 1.6 × 10^10^ M^−1^ · s^−1^; extended conjugation (11 double bonds) limits redox‐sensitive NF‐*κ*B/AP‐1 activation [76–78]	—	Plasma kinetics: *C*_max_ 0.5–1.0 *μ*M at 24–48 h; *t*½ 2–3 days; repeated intake ↑ antioxidant status [86]	Strong (+++)—Robust physicochemical basis and human PK support achievable antioxidant exposure [76–78, 87]
Endothelial barrier and CAMs	In HUVECs: ↓ ICAM‐1 under TNF‐*α*; preserves barrier integrity under LPS/TNF‐*α*; minimal effect on COX‐2/platelet EC adhesion molecules; targets TNF‐*α* (not IFN‐*γ*) pathway [75]	—	Limited early vascular data (tomato‐based interventions) [75]	Moderate (++)—Clear endothelial effects in vitro; translational promise for vascular inflammation [75]
NF‐*κ*B/AP‐1 and cytokine signaling	Inhibits IKK and RelA/p65 nuclear translocation; stabilizes I*κ*B*α* [79]; engages PI3K/Akt → Nrf2/ARE (↑HO‐1, ↑NQO1, ↑GST) [59, 60]; ↓ IL‐8 in smoke‐stimulated THP‐1 macrophages [92]; dose‐dependent ↓ iNOS/NO (~40%) in RAW264.7 [99]	↓ LPS‐driven inflammatory signaling and cell migration [97, 98]	Biomarker changes reported in small cohorts [74, 75]	Moderate (++)—Deep mechanistic support preclinically; early human markers align [71–81, 92, 97–99]
Immune modulation (NK/PBMC)	NK cells: At ~5 *μ*M ↑ viability and cytotoxicity, ↓ caspase‐3/9, ↑ IFN‐*γ*; no change in key NK receptors [92, 93]	—	PBMC: ↑ TNF‐*α* and IL‐1*β* with ↓ IL‐2/IL‐10/IFN‐*γ* (context‐dependent, proinflammatory shift) [94]	Mixed (+/−)—Direction depends on context/dose/cell type; human functional validation needed [92–94]
Respiratory/allergic inflammation	↓ GATA‐3/IL‐4; ↓ eosinophil peroxidase and MMP‐9; ↓ leukocyte infiltration in airway models [100]	Reduced pulmonary inflammatory cell influx in murine allergy models [100]	—	Limited (+)—Consistent preclinical anti‐Th2 signals; no clinical allergy trials yet [100]
Pancreatic protection (acute pancreatitis models)	Pancreatic acinar cells: ↓ ROS and IL‐6; limits NF‐*κ*B activation [96]	—	—	Limited (+)—Strong cellular signal; needs animal and clinical confirmation [75]
Cardiovascular protection/post‐MI remodeling	Vascular/immune cells: ↓ TNF‐*α*–I*κ*B phosphorylation, NF‐*κ*B activation [75]	Post‐MI mouse models: ↓ inflammation and apoptosis; ↓ NF‐*κ*B p65 phosphorylation [29]	—	Moderate (++)—Mechanistic and post‐MI animal support; human outcomes not established [29, 75]
Cancer/angiogenesis	Antiangiogenic at 10 *μ*M with ↑ IFN‐*γ* (~531%) and ↑ IL‐12 (~163%) in HUVEC; ↓ MMP‐9 [95]	Synergy with lutein/*β*‐carotene: ↓ NO, PGE2, superoxide; ↓ iNOS/COX‐2/NADPH oxidase; ↓ TNF‐*α* [98]	—	Moderate (++)—Multiple antiangiogenic/anti‐inflammatory mechanisms preclinically; no preventive RCTs [95, 98]
Epigenetic and microRNA modulation	↓ DNMT activity (~40%) and ↑ HAT (~60%); ↓ DNA methylation at IL‐10/TGF‐*β* promoters; ↑ histone H3 acetylation [92, 93]; ↑ miR‐146a/miR‐155 targeting IRAK1/TRAF6 [84]	—	—	Limited (+)—Emerging mechanistic layer; translational significance pending [82–84]
Bioavailability and processing	—	—	Tomato paste ~2.5× ↑ vs. fresh (cis‐isomerization) [85]; fat coingestion ↑ 3–5× (e.g., 0.3% → 1.5% with olive oil) [63]; PK: *C*_max_ 24–48 h; *t*½ 2–3 days [85]; women > men [88]	Strong (+++)—Consistent human feeding and PK data; supports processed tomato + fat intake [85–88]
Interindividual variability (genetics)	—	—	BCO1 A379V: 40%–60% ↓ lycopene cleavage; higher plasma levels [89]; SCARB1 rs5888: ~25% ↓ absorption; 3–10× variability across individuals [90, 91]	Moderate (++)—Genetic variation materially impacts exposure; consider in trial design [89–91]
HMGB1/DAMP signaling	In HUVEC and in vivo: Inhibits HMGB1 release and HMGB1‐mediated signaling; ↓ TLR‐2/‐4 and RAGE; ↓ CAMs under LPS; reports also note proinflammatory triggers in ECs (context‐dependent) [101]	Confirmatory animal work reported [101]	—	Limited (+)—Direction may vary by context; overall anti‐HMGB1 trend preclinically [101]

*Note:* (+++) strong consistent evidence, (++) moderate evidence, (+) limited evidence, (+/−) mixed/inconclusive evidence.

### 7.3. Fucoxanthin

Fucoxanthin is a type of carotenoid composed of carbon, hydrogen, and epoxy, a functional group. Fucoxanthin is a carotenoid that is naturally found in the cells of various edible brown seaweeds, diatoms, crypto algae, brown algae, and dinoflagellates, as shown in Table 1. Its allene bond and 5,6‐monocyclic oxidation bond define it. Additional research has demonstrated the several positive health advantages of fucoxanthin and its derivatives, particularly the impacts on weight loss, antibacterial, anti‐inflammatory, antioxidant, anticancer, and hypertensive disorders [103–105].

At the molecular level, fucoxanthin′s bioactivity is primarily attributed to its unique structural features, including the presence of an allene bond, acetyl group, and hydroxyl groups that confer superior antioxidant properties compared to other carotenoids. The compound exhibits exceptional singlet oxygen quenching ability with a rate constant of 1.4 × 10^10^ *M* ^−1^ *s* ^−1^, making it 13.5 times more effective than *α*‐tocopherol [106]. Fucoxanthin′s antioxidant mechanism involves the donation of electrons from its conjugated polyene chain to neutralize free radicals, while its epoxy groups participate in hydrogen atom transfer reactions with peroxyl radicals [107].

The anti‐inflammatory activity of fucoxanthin operates through multiple interconnected pathways. The compound directly binds to the p65 subunit of NF‐*κ*B, preventing its nuclear translocation and subsequent transcription of inflammatory genes. This inhibition occurs through fucoxanthin′s ability to stabilize the I*κ*B‐NF‐*κ*B complex by preventing IKK phosphorylation at serine residues 176 and 180 [108]. Additionally, fucoxanthin modulates the mitogen‐activated protein kinase (MAPK) cascade by inhibiting the phosphorylation of extracellular signal‐regulated kinases (ERK1/2), p38 MAPK, and c‐Jun N‐terminal kinase (JNK), thereby reducing the production of proinflammatory mediators including IL‐1*β*, IL‐6, and TNF‐*α* [109].

Fucoxanthin′s anticancer mechanisms involve multiple cellular targets and signaling pathways. The compound induces apoptosis through both intrinsic and extrinsic pathways by upregulating proapoptotic proteins (Bax, Bad, and cytochrome c) while downregulating antiapoptotic proteins (Bcl‐2, Bcl‐xL, and survivin). The intrinsic pathway is activated through mitochondrial membrane depolarization and subsequent release of cytochrome c, leading to caspase‐9 and caspase‐3 activation [110]. Fucoxanthin also modulates cell cycle progression by inducing G0/G1 phase arrest through downregulation of cyclin D1, cyclin‐dependent kinase 4 (CDK4), and retinoblastoma protein (Rb) phosphorylation [111]. Furthermore, the compound inhibits cancer cell metastasis by suppressing matrix metalloproteinase‐2 (MMP‐2) and MMP‐9 expression through the inhibition of NF‐*κ*B and AP‐1 signaling [112] as shown in Figure 4.

Recent studies have revealed that fucoxanthin′s bioactivity is also mediated through epigenetic mechanisms. Treatment with fucoxanthin (50 *μ*M for 48 h) significantly reduces DNA methylation at the promoter regions of tumor suppressor genes, including p16, p21, and BRCA1, while simultaneously decreasing the expression of DNMTs (DNMT1, DNMT3A, and DNMT3B) [113]. The compound also modulates histone modifications by increasing histone H3 lysine 9 acetylation (H3K9ac) and reducing histone H3 lysine 27 trimethylation (H3K27me3) at the promoters of apoptosis‐related genes [114].

The bioavailability of fucoxanthin is significantly influenced by its source, extraction method, and formulation. Native fucoxanthin from brown algae exhibits poor bioavailability due to its hydrophobic nature and susceptibility to degradation. Oral bioavailability of fucoxanthin ranges from 1.8% to 8.9%, depending on the source and processing conditions [115]. However, fucoxanthin undergoes extensive metabolism in the intestine and liver, where it is primarily converted to fucoxanthinol and amarouciaxanthin A by intestinal enzymes, with these metabolites showing higher stability and bioactivity than the parent compound [116].

The absorption of fucoxanthin follows a complex process involving incorporation into mixed micelles in the small intestine, followed by uptake via scavenger receptor class B type 1 (SR‐B1) and cluster determinant 36 (CD36) transporters. The compound is then incorporated into chylomicrons and transported to the liver via the lymphatic system [117]. Fucoxanthin accumulates preferentially in adipose tissue, liver, and kidneys, with tissue distribution being influenced by the presence of carotenoid‐binding proteins and lipoprotein carriers [118].

Recent advances in fucoxanthin formulation have significantly improved its bioavailability. Nanoencapsulation techniques, including liposomal encapsulation and solid lipid nanoparticles, have enhanced fucoxanthin stability and bioavailability by 3–5‐fold compared to conventional formulations [119]. Fucoxanthin‐loaded chitosan nanoparticles demonstrate 4.2‐fold higher bioavailability and 2.8‐fold better cellular uptake compared to free fucoxanthin [120]. Additionally, coadministration with dietary fats increases fucoxanthin absorption by 2.5–3.0‐fold, while the presence of other carotenoids, particularly *β*‐carotene, can competitively inhibit fucoxanthin uptake [121]. Genetic polymorphisms in key metabolic enzymes may significantly affect individual responses to fucoxanthin supplementation. Variants in the BCO1 gene, particularly the rs7501331 polymorphism, result in 30%–45% differences in fucoxanthin metabolism efficiency [122]. These genetic variations contribute to the substantial interindividual variability (4–8‐fold) observed in plasma fucoxanthin and its metabolite concentrations following standardized supplementation [91].

Fucoxanthin, isolated from the diatom *Chaetoceros calcitrans*, has been found to possess an anticancer action, particularly against breast cancer. Further analysis of crude extracts revealed that the concentrated fucoxanthin fraction exhibited potent apoptotic effects on liver cancer cells in vitro. This was achieved by modulating a series of genes involved in cell signaling (ERK1/2, AKT1, JNK), apoptosis (BID, BAX, APAF, Bcl‐2, CYCS), and antioxidants (SOD1, SOD2, CAT). Furthermore, this study revealed fucoxanthin′s anticancer efficacy against colorectal cancer. The findings of their study demonstrated that giving fucoxanthin to male ApcMin/+ mice treated with dextran sodium sulfate (DSS) for 5 weeks dramatically reduced the number of colon adenocarcinomas. The effect of fucoxanthin also decreased the expression of cyclin D1 [29].

Karpiski and Adamczak investigated the antibacterial activity of fucoxanthin versus 13 aerobic and seven anaerobic pathogenic bacteria, and the results obtained showed that fucoxanthin has antibacterial action with MIC ranging from 62.5 to 500 *μ*g/mL [123]. Deyab and Abou‐Dobara showed that fucoxanthin, extracted from *Turbinaria triquetra* brown seaweed (Phaeophyceae), showed antibacterial effects against *Bacillus cereus*, *Bacillus subtilis*, *Escherichia coli*, *Klebsiella pneumoniae*, *Pseudomonas aeruginosa*, and *Staphylococcus aureus*. The inhibition zone ranged from 0.5 to 1.8 mm at a concentration of 10 *μ*g/mL and from 4.0 to 7.0 mm at 100 *μ*g/mL [124]. Rajauria and Abu‐Ghannam demonstrated the antibacterial properties of pure fucoxanthin isolated from the brown alga *Himanthalia elongata* (Phaeophyceae) in the presence of *Listeria monocytogenes*. The inhibition zone was 10.89 mm [125]. In addition, Liu et al. revealed the antimicrobial properties of fucoxanthin. A sample of edible seaweed, *Undaria pinnatifida* (Phaeophyceae), was used to extract the fucoxanthin pigment, which had a purity level of 82.70%. The extracted pigment was then tested against five different human diseases. Fucoxanthin had strong inhibitory effects on the growth of Gram‐positive bacteria *E. faecalis*, *B. subtilis*, *Enterococcus* sp., and *S. aureus*, as controlled by the agar well diffusion technique. The diameters of the inhibitory zones measured 25.24, 25.49, 12.66, and 21.80, respectively [126]. Fucoxanthin may modulate the attenuation of inflammation in the case of infection, mainly induced by Gram‐negative bacteria. The LPS is an endotoxin that is present in the membranes of Gram‐negative bacteria. LPS affects the inflammatory response that occurs during infection, including symptoms such as fever, microbial invasion, and septic shock [108]. Fucoxanthin was demonstrated to suppress the NF‐*κ*B activation and MAPK phosphorylation, therefore inhibiting the production of proinflammatory cytokines (IL‐1*β*, IL‐6, and TNF‐*α*) generated by LPS. Additionally, it decreased the amounts of protein COX‐2 and iNOS [127–129]. Regrettably, it is unknown how fucoxanthin directly inhibits bacteria [108]. The antibacterial and antioxidant properties of natural chemical substances are related, according to the literature [130]. There are three main methods by which antioxidants can exert their antibacterial activity: cytoplasmic leakage, suppression of nucleic acid synthesis, and permeability of the outer membrane [124]. The differential antibacterial impact of fucoxanthin on Gram‐positive bacteria compared to Gram‐negative bacteria implies that the biological activity of this chemical depends on the composition and architecture of the cell walls of these two types of bacteria [123]. Table 4 consolidates the available evidence for fucoxanthin, stratifying findings by experimental model to highlight gaps between preclinical promise and clinical validation.

**Table 4:** Evidence summary for fucoxanthin health outcomes.

Outcome	In vitro evidence	Animal studies	Human studies	Strength of evidence and translational relevance
Antioxidant activity	Strong singlet oxygen quenching (1.4 × 10^10^ *M* ^−1^ *s* ^−1^); better than *α*‐tocopherol; acts via electron donation and H‐atom transfer [106, 107]	—	No direct trials; inferred from dietary algae antioxidant effects	Strong (+++)—Biochemically well characterized; excellent quenching performance supports therapeutic interest
Anti‐inflammatory pathways	Inhibits NF‐*κ*B by stabilizing I*κ*B; ↓ phosphorylation of IKK (Ser176/180); ↓ ERK1/2, JNK, p38 MAPK; ↓ IL‐1*β*, IL‐6, TNF‐*α*; ↓ iNOS, COX‐2 [108, 109, 127–129]	Protective in LPS and DSS‐induced inflammation; reduced cytokine levels in tissues [29, 108]	No clinical trials yet	Moderate (++)—Strong mechanistic evidence; applicable to infection‐ and inflammation‐driven conditions
Anticancer mechanisms	Induces apoptosis (↑ Bax, Bad, cytochrome c; ↓ Bcl‐2, survivin); cell cycle arrest (↓ cyclin D1, CDK4, Rb‐p); ↓ MMP‐2/9 via NF‐*κ*B/AP‐1 inhibition [110–112]	Effective in ApcMin/+ mice: ↓ colorectal tumors, ↓ cyclin D1; inhibits breast/liver tumor markers [29]	No human oncology data	Moderate (++)—Strong in vitro and in vivo; potential as chemopreventive agent
Epigenetic modulation	↓ DNMT1/3A/3B; ↓ DNA methylation at p16, p21, BRCA1 promoters; ↑ H3K9ac, ↓ H3K27me3 [113, 114]	—	—	Limited (+)—Novel mechanism of action; limited to cellular models
Antibacterial activity	Inhibition zones in vitro vs. Gram‐positive bacteria (e.g., *E. faecalis* 25.2 mm, *S. aureus* 21.8 mm); *M* *I* *C* = 62.5–500 *μ* *g*/*m* *L* [123–126]	Not well studied in systemic infection models	No trials	Moderate (++)—Consistent antibacterial activity in vitro; especially against Gram‐positive strains
Antiobesity/metabolism	—	—	Some reported benefits in animal/human combined dietary studies (in other literature, not detailed here)	Limited (+)—Claimed effects in metabolism, but current section lacks direct data
Bioavailability	—	—	Native form: Poor (1.8%–8.9%); ↑ bioavailability with fats (2.5–3×), nanoformulations (3–5×), chitosan NPs (4.2×), ↓ with *β*‐carotene [115–121]	Strong (+++)—Extensive characterization of absorption, metabolism, and formulation strategies
Metabolism and tissue distribution	—	Rapid conversion to fucoxanthinol and amarouciaxanthin A in the intestine/liver; accumulates in adipose, liver, and kidney [116, 118]	Confirmed in animal/human metabolic tracing studies	Moderate (++)—Bioactive metabolites identified; supports indirect effects through derivatives
Genetic variability	—	—	BCO1 rs7501331 polymorphism linked to 30%–45% variation in metabolism; 4–8× interindividual differences [70, 102]	Moderate (++)—Genetic differences influence fucoxanthin metabolism and clinical responsiveness

*Note:* (+++) strong consistent evidence (++), moderate evidence, (+) limited evidence, (+/−) mixed/inconclusive evidence.

### 7.4. Lutein

Lutein is a carotenoid composed of carbon, hydrogen, and hydroxy functional groups, classified specifically as a xanthophyll carotenoid. It has gained significant attention in the medical field due to its diverse biological activities, including anti‐inflammatory, ocular protective, antibacterial, antioxidant, neuroprotective, cardioprotective, antiplasmodial, and antiviral effects [120]. In microalgae such as *Chlorella* sp., *Scenedesmus incrassatulus*, and *Chlamydomonas reinhardtii* (Chlorophyta), lutein plays a crucial role as a key metabolite, intimately involved in photosynthesis, light harvesting, and defense against photooxidative damage. Notably, several microalgae species accumulate high concentrations of lutein (5–10 mg/g), making them promising sources for commercial and therapeutic applications [35].

Recent studies have demonstrated lutein′s ability to inhibit the growth and proliferation of several clinically relevant bacterial species, including *S. aureus*, *Staphylococcus saprophyticus*, *Enterococcus faecium*, *E. coli*, *K. pneumoniae*, and *P. aeruginosa*, at concentrations of 8 and 256 *μ*g/mL. For instance, *E. faecium* and *P. aeruginosa* growth was notably reduced by lutein at concentrations of 8 and 256 *μ*g/mL, respectively, while the minimum inhibitory concentration (MIC) for *K. pneumoniae*, *E. coli*, and *S. aureus* was found to be 32 *μ*g/mL [35, 130]. These findings suggest that lutein holds potential as a natural antibacterial agent. Although the precise mechanism of its antibacterial action is still being elucidated, it has been hypothesized that lutein may aid in the accumulation of lysozyme, an immunological enzyme involved in the breakdown of bacterial cell walls.

Recent research has provided further insight into the biochemical mechanisms underlying lutein′s health‐promoting effects. Notably, lutein has been shown to disrupt bacterial quorum sensing (QS) and biofilm formation, particularly in *P. aeruginosa*, by interfering with the expression of key QS‐regulated genes such as lasB, rhlA, rhlR, lasR, and vfr. This disruption results in reduced production of virulence factors like pyocyanin, elastase, and rhamnolipids and can even enhance the efficacy of antibiotics like tobramycin against biofilm‐encapsulated bacteria [131]. Additionally, lutein′s potent antioxidant activity allows it to neutralize ROS, modulate inflammatory signaling pathways such as NF‐*κ*B and MAPK, and upregulate the Nrf2 pathway, thereby enhancing cellular antioxidant defenses [132, 133]. These actions collectively contribute to its anti‐inflammatory and tissue‐protective effects.

The efficacy and bioavailability of lutein are significantly influenced by its source and chemical form. Lutein derived from eggs is generally more bioavailable than that from vegetables or supplements, likely due to the lipid‐rich matrix of eggs facilitating better micelle formation and intestinal absorption [134]. Oil‐based supplements also offer higher plasma lutein peaks compared to vegetables, while the bioavailability from leafy greens like spinach is superior to that from cruciferous vegetables such as broccoli, largely due to differences in food matrix and preparation methods [135]. Furthermore, there is ongoing debate regarding whether free or esterified lutein is more efficiently absorbed, with some studies favoring free lutein and others suggesting that esterified forms may be advantageous under certain conditions [136]. The progression of lutein research from laboratory to clinical settings is mapped in Table 5, which organizes evidence according to study design and outcome measures.

**Table 5:** Evidence summary for lutein health outcomes.

Outcome	In vitro evidence	Animal studies	Human studies	Strength of evidence and translational relevance
Antioxidant activity	Potent ROS scavenger; activates Nrf2 pathway; suppresses oxidative stress via modulation of NF‐*κ*B and MAPK [132, 133]	Protection against oxidative damage in neural and ocular tissues (referenced in other literature)	Improved antioxidant status in plasma in egg/lutein dietary trials	Strong (+++)—Supported by cellular, animal, and human studies with nutritional interventions [132, 133]
Anti‐inflammatory effects	Inhibits NF‐*κ*B and MAPK activation; ↓ proinflammatory cytokines; enhances cellular defenses via Nrf2/HO‐1 pathway [132]	Reduced tissue inflammation in neurodegeneration and eye models (literature‐supported)	Associated with ↓ inflammation markers in dietary intervention studies	Moderate (++)—Strong preclinical evidence; modest clinical support, especially for eye and vascular health [132]
Antibacterial activity	Inhibits growth of *S. aureus*, *S. saprophyticus*, *E. faecium*, *E. coli*, *K. pneumoniae*, *P. aeruginosa*; MICs: *E. faecium* (8 *μ*g/mL), *P. aeruginosa* (256 *μ*g/mL), *K. pneumoniae*/*E. coli*/*S. aureus* (32 *μ*g/mL); hypothesized lysozyme accumulation mechanism [35, 130]	—	—	Moderate (++)—Direct antimicrobial effect shown in vitro; requires animal and human validation [35, 130]
Quorum sensing and biofilms	Inhibits lasB, rhlA, rhlR, lasR, and vfr in *P. aeruginosa*; ↓ pyocyanin, elastase, rhamnolipids; ↑ tobramycin efficacy against biofilm‐encapsulated bacteria [131]	—	—	Limited (+)—Mechanistically significant; currently limited to microbial and molecular models [131]
Neuroprotective effects	—	Evidence in animal models of brain ischemia and retinal protection (referenced in external literature)	Associated with improved cognitive and visual function in older adults in some cohort studies (external literature)	Moderate (++)—Widely studied in neuro‐ocular models; promising cognitive outcomes in humans
Ocular health	—	Protection against blue light and photooxidative retinal damage (animal and cell studies in literature)	Well‐studied: ↑ macular pigment density, ↓ risk of AMD with lutein‐rich diets or supplementation [133]	Strong (+++)—Clinically validated in age‐related macular degeneration (AMD) and vision preservation [133]
Bioavailability	—	—	Egg‐derived lutein > vegetables/supplements due to lipid matrix facilitating micelle formation and absorption [134]; oil‐based supplements > vegetables for plasma peaks [135]; leafy greens (spinach) > cruciferous vegetables (broccoli) due to food matrix differences [135]; ongoing debate: Free vs. esterified lutein absorption efficiency [136]	Strong (+++)—Extensive human feeding studies demonstrate source‐dependent bioavailability differences [134–136]

*Note:* (+++) strong consistent evidence, (++) moderate evidence, (+) limited evidence, (+/−) mixed/inconclusive evidence.

### 7.5. Astaxanthin

Astaxanthin is a type of carotenoid composed of carbon, hydrogen, and a keto‐a functional group. Astaxanthin is a carotenoid of xanthophyll that is identified in seafood such as shrimp and salmon. With its exceptional antifibrogenic properties, astaxanthin is a naturally occurring red–orange carotenoid that is lipid soluble. Astaxanthin is increasingly sought after as a multitarget pharmacological treatment against a variety of disorders due to its strong antioxidant properties and anti‐inflammatory, antiapoptotic, and immune‐modulatory effects [137]. Astaxanthin demonstrates exceptional antioxidant capacity, with studies showing it has 100–500 times higher oxygen radical absorbance capacity (ORAC) than *α*‐tocopherol (vitamin E) and 10 times higher free radical inhibitory activity than related antioxidants [138]. This superior antioxidant potency is attributed to its unique molecular structure, which allows it to span cellular membranes with polar hydroxyl groups anchored at membrane surfaces while its conjugated polyene chain extends across the lipid bilayer, enabling neutralization of both lipophilic and hydrophilic ROS [139]. Mechanistically, astaxanthin exerts its biological effects through multiple interconnected pathways. At the molecular level, it inhibits NF‐*κ*B activation, thereby reducing expression of proinflammatory cytokines including TNF‐*α*, IL‐1*β*, and IL‐6. Additionally, it modulates COX‐2 and lipoxygenase pathways, resulting in decreased prostaglandin E2 and leukotriene synthesis [140]. In metabolic regulation, astaxanthin activates peroxisome proliferator‐activated receptor‐*γ* coactivator‐1*α* (PGC‐1*α*) and adenosine monophosphate‐activated protein kinase (AMPK), enhancing mitochondrial biogenesis and promoting fatty acid oxidation over carbohydrate metabolism [141] as shown in Figure 4.

Astaxanthin may be able to stop quiescent hepatic stellate cells from becoming active and from activating activated hepatic stellate cells back into a quiescent state. In the liver, astaxanthin decreases collagen accumulation while also lowering the expression of fibrogenic genes. The protective impact of astaxanthin against the development of liver fibrosis may be due to its anti‐inflammatory properties and its potential to increase antioxidant properties. Additionally, lycopene has been demonstrated to reverse the course of nonalcoholic steatohepatitis in mice and decrease hepatic steatosis [18, 142]. However, the therapeutic potential of astaxanthin is significantly limited by its poor oral bioavailability. The oral bioavailability of astaxanthin ranges around 10%–50% of the given dose as a result of its poor solubility in water and poor absorption by epithelial cells [143]. After ingestion, astaxanthin is combined with bile acid in the intestine, forming micelles. Intestinal mucosal cells absorb astaxanthin from micelles, which are then incorporated into chylomicra and released into the lymphatic system before entering systemic circulation [144]. The bioavailability varies significantly based on formulation strategies, with lipid‐based formulations showing substantial improvements. Studies have demonstrated that proprietary lipid‐based astaxanthin formulations can achieve 3.6‐fold greater bioavailability compared to unformulated astaxanthin oil [145]. Advanced delivery systems, including nanoemulsions, liposomes, solid lipid nanoparticles, and polymeric nanoparticles, have been developed to overcome these absorption limitations and enhance clinical efficacy [146].

*In vitro*, astaxanthin exhibits significant antibacterial action against isolates of *B. cereus*, *P. aeruginosa,* and *E. coli,* with an MIC value of 16 *μ*g/mL, while the reference standard strain is in the range of 0.25–0.125 *μ*g/mL. However, astaxanthin′s MIC against *S. aureus* was less than that of novobiocin. Furthermore, astaxanthin exhibited the same MBC value as novobiocin against *B. cereus*, *E. coli*, and *P. aeruginosa*; this value was less than that of ciprofloxacin against all bacterial strains examined [19]. Astaxanthin has shown potent inhibitory effects, namely, on the topoisomerase IV subunits ParC and ParE, in silico. These effects were validated, and the possible association of oxidative stress with the bacterial lethality of astaxanthin was explored using in vitro tests. Multiple studies have recorded the antimicrobial characteristics of astaxanthin, providing an indication of its ability to inhibit the growth of bacteria in a dosage‐dependent way, as well as its ability to kill both Gram‐positive and Gram‐negative bacteria [147–150]. Aribisala et al. provide evidence that astaxanthin is effective against both Gram‐positive and Gram‐negative organisms. Their study also demonstrates that the time‐kill sensitivity test of astaxanthin reveals a reduction in bacterial viability that is dependent on concentration [151]. In particular, astaxanthin exhibited a lower MIC against *S. aureus* compared to novobiocin in this investigation. This discovery corroborates the in silico findings, which indicated that astaxanthin exhibited a greater attraction toward the topo2A druggable targets (topoisomerase IV ParC/ParE) in Gram‐positive organisms compared to Gram‐negative targets (GyrA/GyrB). To assess the clinical relevance of astaxanthin′s reported bioactivities, Table 6 categorizes evidence by research level, distinguishing mechanistic insights from human health outcomes.

**Table 6:** Evidence summary for astaxanthin health outcomes.

Outcome	In vitro evidence	Animal studies	Human studies	Strength of evidence and translational relevance
Antioxidant capacity	ORAC 100–500× *α*‐tocopherol; ~10× higher free‐radical inhibition vs. related antioxidants; membrane‐spanning quenching (polar heads + polyene core) [137, 138]	Reduces oxidative stress markers; protects lipids/proteins in tissues (general preclinical)	—	Strong (+++)—Exceptional physicochemical antioxidant profile [137, 138]
Anti‐inflammatory (NF‐*κ*B/eicosanoids)	Inhibits NF‐*κ*B; ↓ TNF‐*α*, IL‐1*β*, IL‐6; modulates COX‐2/LOX → ↓ PGE_2_, leukotrienes [139]	Confirms ↓ inflammatory signaling in models (general preclinical)	—	Moderate (++)—Consistent mechanistic data; clinical validation pending [139]
Mitochondrial and metabolic regulation	Activates PGC‐1*α* and AMPK → ↑ mitochondrial biogenesis; shifts to fatty acid oxidation [140]	Improved metabolic markers in vivo (supporting preclinical)	—	Moderate (++)—Strong mechanism; translational promise in metabolic disease [140]
Antifibrotic/liver protection	Prevents HSC activation; may revert activated HSCs; ↓ collagen and fibrogenic genes; protects against liver fibrosis [18, 141]	Antifibrotic effects in NASH/steatosis models [123, 124]	—	Moderate (++)—Compelling liver antifibrotic signals preclinically [18, 141]
Antibacterial activity	MIC ~16 *μ*g/mL vs. *B. cere*us, *P. aeruginosa*, and *E. coli*; time‐kill, dose‐dependent effects; topo IV (ParC/ParE) targeting supported in silico; active vs. Gram+ and Gram− [146, 151]	—	—	Moderate (++)—Reproducible in vitro efficacy; mechanism supported [146, 151]
Bioavailability and formulations	—	—	Oral bioavailability ~10%–50% due to low solubility; bile‐micelle uptake → chylomicrons → lymph [125, 126]; lipid formulations ↑ 3.6× vs. unformulated [144]; nanoemulsions/liposomes/SLN/polymeric NPs enhance delivery [145]	Strong (+++)—PK challenges known; formulation solutions well documented [142, 145]

*Note:* (+++) strong consistent evidence, (++) moderate evidence, (+) limited evidence, (+/−) mixed/inconclusive evidence.

### 7.6. Canthaxanthin

Canthaxanthin is a type of carotenoid composed of carbon, hydrogen, and a keto functional group. The red keto‐carotenoid canthaxanthin is found abundantly in various bacteria and algae, including *Graesiella emersonii* and *Desmodesmus abundans* (formerly *Chlorella fusca*) (Chlorophyta). However, the majority of commercially available canthaxanthin is produced synthetically. Its biosynthesis proceeds from *β*‐carotene via the action of a single enzyme, *β*‐carotene ketolase, which introduces carbonyl groups at positions 4 and 4 ^′^ of the *β*‐carotene molecule. The biosynthetic pathway starts from acetyl‐CoA and progresses through phytoene, lycopene, *β*‐carotene, and canthaxanthin, before undergoing further oxidation to form astaxanthin. This mechanistic insight has enabled the development of optimized microbial production platforms for commercial purposes [152, 153].

Canthaxanthin is allowed to be used in broiling chicken and salmonid fish feed in the United States but only at concentrations below 30 mg/0.45 kg of solid or semisolid feed or below 30 mg/0.74 L of liquid food. However, in the European Union, canthaxanthin can only be used in food products at a maximum content of 25 mg/kg of final food. Canthaxanthin is permitted to be used as a food additive according to the law [154]. Canthaxanthin has been shown to have antioxidant and antiaging qualities, making it effective in removing free radicals, minimizing oxidative stress, and improving the body′s natural antioxidant defenses [128]. In vitro research by Esatbeyoglu et al. has shown that canthaxanthin, a ketocarotene, has superior antioxidant and free radical scavenging properties in comparison with carotenoids such as lycopene or *β*‐carotene [155]. The coupling of the keto group with the polyene backbone increases stability and increases the ability to stabilize radicals with a carbon center.

When the conjugated keto‐carotenoids, either astaxanthin or canthaxanthin, are added to rat liver microsomes undergoing radical‐initiated lipid peroxidation under air, they are as effective as alpha‐tocopherol in inhibiting this process, which demonstrates their potent membrane‐protective antioxidant activity [156]. The structural configuration of canthaxanthin, with its symmetrical keto groups at positions 4 and 4 ^′^ of the *β*‐carotene molecule, enables enhanced radical scavenging capacity compared to non–keto‐carotenoids [142]. Recent research has revealed that canthaxanthin′s effects were mediated by the SIRT6 pathway, highlighting its potential as a therapeutic agent for liver fibrosis and cancer. The SIRT6 pathway represents a novel molecular mechanism through which canthaxanthin exerts its cellular protective effects, linking its antioxidant properties to epigenetic regulation of cellular aging and stress response [157].

The bioavailability of canthaxanthin is influenced by several factors, including coadministration with other carotenoids and formulation approaches. Ingestion of a combined pharmacologic dose of beta‐carotene and canthaxanthin reduces the bioavailability of the canthaxanthin dose, indicating competitive absorption mechanisms between carotenoids that may limit therapeutic efficacy when multiple carotenoids are consumed simultaneously [158]. As a lipophilic compound, canthaxanthin faces similar bioavailability challenges to other carotenoids. The development of encapsulation technologies and improved delivery systems has become increasingly important for enhancing its therapeutic potential [159]. Recent advances in microbial production have also focused on improving the stability and bioavailability of naturally produced canthaxanthin compared to synthetic alternatives [152].

Furthermore, Okai et al. have demonstrated that canthaxanthin possesses immunomodulatory properties by promoting the growth and function of mouse immunocompetent cells [60]. Canthaxanthin supplementation increased T and B lymphocyte proliferation in rat spleen [160]. Furthermore, canthaxanthin treatment has been reported to improve mitogen‐induced lymphocyte proliferation, even without demonstrating provitamin action. Treatment with canthaxanthin improved the expression of activation markers for T‐helper and natural killer cells in human peripheral blood mononuclear cells. Canthaxanthin has been shown to be effective in the treatment of tanning and photosensitive disorders [161].

The anticancer and cancer‐fighting behaviors of canthaxanthin may be assigned to its radical trapping or chain‐breaking processes, as supported by evidence [162]. This keto‐carotenoid triggered a decrease in oral carcinogenesis in F344 rats by inhibiting cell proliferation and reducing polyamine levels in oral mucosal tissues [163]. However, it has been shown that women with cervical cancer have reduced levels of canthaxanthin in their plasma. Canthaxanthin has shown efficacy in delaying the initiation of dimethylbenzanthracene‐induced breast cancer in female Sprague‐Dawley rats. However, it does not appear to have any noticeable effect on the development of methyl nitrosourea‐induced carcinogenesis of the same kind [28, 160]. Table 7 provides an integrated overview of canthaxanthin evidence, comparing in vitro mechanisms, animal model findings, and human clinical data.

**Table 7:** Evidence summary for canthaxanthin health outcomes.

Outcome	In vitro evidence	Animal studies	Human studies	Strength of evidence and translational relevance
Sources and biosynthesis	Keto‐carotenoid; microbial/algal sources; *β*‐carotene → canthaxanthin via *β*‐carotene ketolase; platform for microbial production [141, 142]	—	—	Moderate (++) = scalable biosynthesis pathways [141, 142]
Regulatory use limits	—	—	Use limits in US/EU feeds/foods (≤ 25–30 mg/kg depending on matrix) [153]	Moderate (++)—Established regulatory framework [153]
Antioxidant/membrane protection	Superior radical scavenging vs lycopene/*β*‐carotene in vitro; keto groups enhance stability; inhibits lipid peroxidation in liver microsomes (≈*α*‐tocopherol efficacy) [155, 156]	—	—	Strong (+++)—Robust antioxidant and chain‐breaking evidence [155, 156]
SIRT6/antifibrotic potential	SIRT6‐mediated protection; links antioxidant effects to epigenetic/stress responses [157]	—	—	Limited (+)—Mechanistic novelty; needs in vivo/human data [157]
Immunomodulation	Promotes growth/function of immunocompetent cells; ↑ T/B cell proliferation; ↑ activation markers in PBMCs [60, 161, 162]	↑ splenic lymphocyte proliferation (rats) [160]	Use in photosensitive disorders/tanning [161]	Moderate (++)—Consistent immune effects; clinical applications niche [49, 144, 145]
Anticancer/chemoprevention	Radical‐trapping mechanisms; ↓ oral carcinogenesis (F344), ↓ polyamines [162, 163]; mixed efficacy across models [160–164]	Multiple rodent models [160, 163, 164]	Plasma levels lower in cervical cancer patients [160]	Moderate (++)—Preclinical support; human causal data limited [160, 162–164]
Bioavailability and delivery	Competitive absorption with *β*‐carotene reduces canthaxanthin bioavailability [158]; encapsulation/delivery systems to improve uptake [159]	—	—	Moderate (++)—Known competition; tech solutions emerging [158, 159]

*Note:* (+++) strong consistent evidence, (++) moderate evidence, (+) limited evidence, (+/−) mixed/inconclusive evidence.

### 7.7. Halocynthiaxanthin

Halocynthiaxanthin is a unique acetylenic carotenoid that can be separated from sea squirts, mainly *Halocynthia roretzi* (Chordata), characterized by its complex molecular structure containing a keto group, acetylenic bonds, a hydroxyl group, and an epoxy group. This structural complexity contributes to its distinctive biological properties and therapeutic potential, making it one of the most bioactive carotenoids discovered to date [164]. The bioavailability of halocynthiaxanthin is influenced by its unique acetylenic structure, which differs significantly from conventional carotenoids. Dietary halocynthiaxanthin can be absorbed from the intestine and found in the plasma as both all‐trans and cis‐isomer forms. Importantly, halocynthiaxanthin is a metabolite of fucoxanthin found mainly in sea squirts; however, mammals cannot convert fucoxanthin to halocynthiaxanthin, indicating that direct dietary intake is necessary for bioavailability in humans. The absorption process involves intestinal uptake mechanisms that allow for the incorporation of both geometric isomers into systemic circulation. The acetylenic bonds in halocynthiaxanthin may affect its stability during digestion and influence its absorption efficiency compared to other carotenoids, while the presence of both polar (hydroxyl, epoxy) and nonpolar regions in the molecule affects its solubility properties and subsequent bioavailability [164]. Halocynthiaxanthin demonstrates the most potent ability to control the generation of free radicals among carotenoids tested through multiple antioxidant mechanisms. Its antioxidant action involves direct radical scavenging through its extended conjugated system with acetylenic bonds, which provides enhanced electron‐donating capacity. Additionally, the keto and epoxy groups contribute to singlet oxygen deactivation, while the hydroxyl groups enable interaction with lipid radicals in membrane systems, effectively inhibiting lipid peroxidation. These multiple antioxidant pathways work synergistically to provide superior protection against oxidative stress compared to conventional carotenoids [164, 165]. Halocynthiaxanthin exhibits comprehensive anticancer mechanisms that operate through several distinct pathways. Halocynthiaxanthin inhibits the activity of tumor promoters in Raji cells that activate the Epstein‐Barr virus (EBV), with this cell line serving as a primary test to assess the efficacy of compounds in preventing tumor promotion [166, 167]. The compound significantly increases DNA fragmentation in cancer cells through activation of apoptotic pathways, involving caspase activation cascades, mitochondrial membrane potential disruption, and enhanced proapoptotic protein expression. Furthermore, halocynthiaxanthin interferes with cell cycle progression in cancer cells, particularly affecting G1/S checkpoint regulation, cyclin‐dependent kinase activity, and DNA synthesis inhibition. At the cellular level, halocynthiaxanthin′s effects are mediated through several key pathways, including activation of Nrf2‐mediated antioxidant response, modulation of mitochondrial electron transport chain function, interference with growth factor signaling cascades, and regulation of genes involved in cell proliferation and apoptosis. These diverse mechanisms allow the compound to target multiple cellular processes simultaneously, resulting in synergistic therapeutic effects that exceed those of simpler carotenoids [168, 169].

Halocynthiaxanthin inhibited the development and spread of HL‐60 human leukemia cells in a manner that depended on both the duration of exposure and the dosage, demonstrating superior efficacy compared to conventional carotenoids due to its unique structural features. The compound, derived from *Halocynthia roretzi*, exhibits antiproliferative and apoptosis‐inducing effects across multiple cancer types, including human leukemia, colon cancer cells, and breast cancer. Halocynthiaxanthin significantly increased DNA fragmentation in HL‐60, MCF‐7, and Caco‐2 cells, demonstrating broad‐spectrum anticancer activity with varying efficacy among different cancer cell lines. The highest sensitivity is observed in hematological cancers, particularly leukemia cells, while moderate to high efficacy is seen in hormone‐dependent breast cancer cell lines and significant activity against colorectal carcinoma cells [170].

The unique combination of functional groups in halocynthiaxanthin contributes to its superior bioactivity through distinct structure–activity relationships. The acetylenic bonds enhance radical scavenging capacity, the keto group facilitates cellular uptake and membrane interaction, the hydroxyl group increases water solubility and bioavailability, and the epoxy group contributes to chemical stability and biological activity. This multifunctional structure allows halocynthiaxanthin to interact with multiple cellular targets simultaneously, resulting in synergistic therapeutic effects that position it as a promising candidate for cancer prevention and treatment applications [171, 172]. Given the limited clinical data for halocynthiaxanthin, Table 8 synthesizes available evidence across experimental platforms to contextualize its therapeutic potential.

**Table 8:** Evidence summary for halocynthiaxanthin health outcomes.

Outcome	In vitro evidence	Animal studies	Human studies	Strength of evidence and translational relevance
Structure and sources	Acetylenic xanthophyll from *Halocynthia roretzi*; keto, acetylenic, hydroxyl, epoxy groups [164]	—	—	Moderate (++)—Distinct structural features [149]
Antioxidant mechanisms	Superior radical control via extended conjugation + acetylenic bonds; keto/epoxy for singlet‐oxygen deactivation; hydroxyls engage lipid radicals [164, 165]	—	—	Moderate (++)—Strong mechanistic basis [164, 165]
Antitumor promotion and apoptosis	Inhibits EBV tumor promoters (Raji assay) [166, 167]; induces apoptosis, disrupts *ΔΨ*m, activates caspases; cell cycle effects (G1/S, CDKs) [168, 170]	—	—	Moderate (++)—Broad in vitro anticancer activity [166, 167]
Signaling pathways	Activates Nrf2; modulates ETC; interferes with growth‐factor signaling; regulates proliferation/apoptosis genes [168, 169]	—	—	Limited (+)—Cellular pathway mapping; needs in vivo
Bioavailability	Absorbed as all‐trans/cis isomers; metabolite of fucoxanthin in sea squirts, but mammals cannot convert fucoxanthin → halocynthiaxanthin; direct intake required [164]	—	—	Limited (+)—Human uptake plausible; dietary source required [164]

*Note:* (+++) strong consistent evidence, (++) moderate evidence, (+) limited evidence, (+/−) mixed/inconclusive evidence.

### 7.8. Peridinin

Peridinin is a unique marine carotenoid structurally characterized by an allenic bond, an epoxide, and a lactone ring within its C37 apocarotenoid backbone. Unlike most carotenoids, whose backbone typically comprises 40 carbon atoms, peridinin′s unusual structure supports its distinctive physicochemical and biological properties. The presence of these functional groups has profound effects on its biochemical behavior, photoprotective roles, and interaction with cellular targets, distinguishing it as a light‐harvesting pigment predominantly found in *Heterocapsa triquetra* (formerly *Heterocapsa triquetra*) (Dinophyceae) and as a compound of high interest in therapeutic and cosmetic innovation. In the DLD‐1 colorectal adenocarcinoma cell line, peridinin is capable of triggering apoptosis [173]. At the molecular and cellular level, peridinin′s mechanisms of action are intricately tied to its structure. Its conjugated polyene system, equipped with lactone and an allenic bond, grants exceptional radical scavenging and singlet oxygen quenching ability. This function is crucial not only for the protection of the photosynthetic apparatus in dinoflagellates by quenching the chlorophyll triplet state and preventing photodamage but also for its potential anticancer activity in mammalian systems [174]. Upon cellular uptake, peridinin is capable of integrating into lipid bilayers, where its structural features facilitate efficient energy transfer and modulate membrane‐associated signaling pathways [173].

In experimental cancer models, such as the DLD‐1 colorectal adenocarcinoma line, peridinin has demonstrated the ability to induce apoptosis. This is mechanistically linked to the upregulation of key proapoptotic enzymes (caspase‐8 and caspase‐9) and the occurrence of chromatin fragmentation after prolonged exposure, which underscores peridinin′s role in disrupting cellular proliferation and survival. Additionally, peridinin interferes with inflammatory and oncogenic signaling mediated by NF‐*κ*B. Biochemical studies show that peridinin inhibits Akt‐mediated activation of IKK, with a subsequent reduction in NF‐*κ*B activation and the expression of its downstream gene products important for both cancer progression and inflammatory responses. These regulatory effects are further supported by in vivo evidence from animal models, where peridinin administration not only suppresses tumor growth but also enhances tumor apoptosis, all without apparent toxicity [173]. Peridinin′s efficacy also extends to T cell lines infected with HTLV‐1, demonstrating a dose‐dependent decline in cell viability and proliferation. This broadens its potential as an antitumor and antiviral agent. Its anti‐inflammatory action emerges through the suppression of delayed‐type hypersensitivity (DTH) responses and by reducing eosinophil migration and eotaxin production, key markers in allergic and inflammatory conditions. Together, these mechanisms highlight peridinin′s role as both a cytoprotective and immunomodulatory agent [175]. Ishikawa et al. found that peridinin hampers IKK activation by reducing Akt activation, as shown by their results [175]. Peridinin demonstrated a substantial reduction in the development of subcutaneous ATL xenografts during vivo trials when administered at a dose of 8.5 mg/kg body weight. Significantly, the level of apoptosis was found to increase in tumors extracted from mice administered peridinin. The results also indicated that the mice treated with the specified dose did not show any signs of toxicity or obvious indications of disease. The results highlight the safety and anti‐ATL properties of peridinin. Additional research is necessary to estimate the lasting impacts and safety of peridinin with respect to AT produced experimentally [37]. Peridinin has anti‐inflammatory properties. Peridinin suppressed DTH responses in mice. Furthermore, peridinin decreased the number of eosinophils in both peripheral blood and ear tissues. Topical application of peridinin reduced both the migration of eosinophils towards eotaxin and the production of eotaxin in ears [176].

The bioavailability of peridinin differs from other carotenoids due to its amphipathic nature: the polar functional groups allow improved integration into protein complexes, such as the peridinin‐chlorophyll‐protein (PCP) complex in dinoflagellates. In this environment, peridinin orientation and interaction maximize excitation energy transfer and protect chlorophyll from oxidative stress [177]. However, in polar biological environments, peridinin′s excited‐state dynamics are altered: increased solvent polarity reduces its fluorescence quantum yield and excited‐state lifetime, likely due to the formation of an intramolecular charge transfer state, which affects how efficiently it transfers energy or exerts antioxidant effects in different cellular contexts [173]. Nonetheless, these properties make peridinin particularly effective in aqueous systems compared to typical hydrophobic carotenoids, potentially enhancing its efficacy and tissue distribution when used pharmaceutically. Structurally, peridinin′s distinctive arrangement around central chlorophyll molecules in protein complexes also allows for nearly complete energy transfer and maximized photoprotection. In contrast to simpler carotenoids, its ability to act both as a light harvester and a photoprotective agent underlines not only its biological significance in marine organisms but also its promise as a robust therapeutic and cosmetic ingredient [177]. The evidence base for peridinin is systematically organized in Table 9, facilitating comparison between mechanistic understanding and clinical translatability.

**Table 9:** Evidence summary for peridinin health outcomes.

Outcome	In vitro evidence	Animal studies	Human studies	Strength of evidence and translational relevance
Structure/function	Allenic bond, epoxide, lactone ring; C37 apocarotenoid; potent radical scavenger/singlet‐oxygen quencher; integrates in membranes [173, 174]	—	—	Moderate (++)—Distinct structure underpins activity [173, 174]
Anticancer (apoptosis)	In DLD‐1 cells: Induces apoptosis (↑ caspase‐8/9, chromatin fragmentation) [173]	Suppresses tumor growth; ↑ tumor apoptosis; no overt toxicity at 8.5 mg/kg in ATL xenografts [37]	—	Moderate (++)—Strong preclinical anticancer signals [158, 161]
NF‐*κ*B/Akt‐IKK axis	Inhibits Akt‐mediated IKK → ↓ NF‐*κ*B activation; downregulates downstream inflammatory/oncogenic genes [175]	Supports anti‐inflammatory/anticancer effects in vivo [37]	—	Moderate (++)—Convergent pathway evidence [37, 175]
Immunomodulation/antiallergy	Suppresses DTH; ↓ eosinophils and eotaxin production; anti‐inflammatory in mice [176]	Confirmed DTH suppression and eosinophil effects [176]	—	Moderate (++)—Translational potential in allergy/inflammation [176]
Bioavailability/photophysics	Amphipathic; efficient energy transfer in PCP complex; solvent polarity alters excited‐state dynamics/ICT; effective in aqueous systems [173, 177]	—	—	Limited (+)—Biophysical advantages; pharmacokinetics in humans not defined [173, 177]

*Note:* (+++) strong consistent evidence, (++) moderate evidence, (+) limited evidence, (+/−) mixed/inconclusive evidence.

### 7.9. Zeaxanthin

Zeaxanthin is a yellow pigment carotenoid composed of carbon, hydrogen, and hydroxyl groups, found across diverse microalgae including heterokonts, rhodophytes, chlorophytes (green algae), cyanobacteria, *Dunaliella salina* (Chlorophyta), *Spirulina* (Cyanobacteria), and *Chlorella* sp. (Chlorophyta), as well as red macroalgae (Corallinaceae: 6–15 *μ*g/g) and microalgae such as *Porphyridium purpureum* (formerly *Porphyridium cruentum*) (Rhodophyta) (0.06–0.27 mg/g) and *Porphyridium cruentum* (1.1 mg/g) [177]. This compound contributes to nature′s yellow coloration and is utilized in foods as a yellow pigment and supplement to help prevent AMD [38, 178].

Biochemically, zeaxanthin accumulates in the retinal macular pigment, where it filters high‐energy blue light (400–500 nm) and neutralizes singlet oxygen and free radicals, thereby protecting retinal cells from oxidative stress, mitochondrial dysfunction, apoptosis, and inflammation, processes implicated in AMD, cataracts, and diabetic retinopathy [126]. Zeaxanthin also operates through mechanisms like electron transfer, hydrogen atom transfer, and radical subtraction to scavenge reactive species [178]. Beyond the eye, its potent radical‐scavenging properties support systemic health: it may act as an indirect antimalarial or parasitism marker and exhibits antioxidant, anti‐inflammatory, antidiabetic, and antiapoptotic effects that underpin neuroprotective potential [179, 180]. Compared to nonpolar carotenoids such as *β*‐carotene and lycopene, zeaxanthin′s polarity enhances its activity in aqueous environments like plasma. Indeed, under oxidative challenge (sunlight and methylene blue), zeaxanthin levels in plasma decline more than those of other carotenoids, indicating heightened reactivity and antioxidant function in the water phase [178].

Bioavailability differences significantly impact efficacy. Studies comparing free versus esterified forms show higher serum responses and increased AUC by ~17% for free lutein versus esters in some trials, while others find diesters to be more effective, depending on formulation and administration context [181]. Novel formulations achieve even greater enhancements: an LZO formulation yielded approximately 1.8× higher *C*_max_ and 2.2× greater AUC_0–72_ for zeaxanthin versus a standard control [182]. In addition, a 6‐month randomized trial demonstrated that micellar diacetate versions of lutein, zeaxanthin, and meso‐zeaxanthin significantly boosted serum zeaxanthin and meso‐zeaxanthin levels compared to traditional forms, highlighting improved bioavailability through micellar emulsions [183]. Another study further corroborates enhanced plasma uptake with newly optimized delivery systems [184].

Dietary sources also exert influence: eggs, rich in fat, improve the bioavailability of zeaxanthin relative to plant matrices. Typical Western diets exhibit a lutein‐to‐zeaxanthin ratio of about 5:1 to 8:1, with an overall intake of both carotenoids generally below the 6–14 mg/day often recommended to reduce the risk of AMD and cataracts [185]. Food and supplement absorption rates vary, with bioavailability from vegetables ranging between 45% and 67% of supplemental crystalline forms [181]. Table 10 presents a hierarchical evidence summary for zeaxanthin, ranking findings from cellular studies through human trials to indicate research maturity.

**Table 10:** Evidence summary for zeaxanthin health outcomes.

Outcome	In vitro evidence	Animal studies	Human studies	Strength of evidence and translational relevance
Sources and distribution	Broad algal sources; quantities noted across taxa [177]	—	—	Moderate (++)—Abundant dietary/microalgal availability [177]
Ocular/macular protection	Filters blue light (400–500 nm); neutralizes singlet O_2_/ROS; protects against mitochondrial dysfunction/apoptosis/inflammation [135, 178]	Retinal protection in models (supporting literature)	Used to help prevent AMD; macular pigment support [164, 165]	Strong (+++)—Cornerstone of macular health; widely applied [38, 135, 178]
Systemic antioxidant/anti‐inflammatory	Radical scavenging via electron transfer, H‐atom transfer, radical subtraction [178]; neuroprotective/antidiabetic/antiapoptotic potential [179, 180]	—	—	Moderate (++)—Mechanistic breadth beyond eye [179, 180]
Bioavailability—Form and matrix	Free vs. esterified: Mixed results; formulation‐dependent [81, 182]; advanced LZO ~1.8× *C*_max_ and 2.2× AUC_0–72_ [181]; improved plasma uptake with optimized systems [185]	—	Eggs (fat matrix) enhance uptake; typical intake below 6–14 mg/day suggested for AMD/cataracts risk reduction; veg bioavailability ~45%–67% of crystalline forms [180, 185]	Strong (+++)—Clear matrix/formulation effects; practical guidance supported [181, 185]
Oxidative challenge in plasma	Higher reactivity in aqueous phase—Zeaxanthin declines faster than other carotenoids under photo‐oxidative stress [178]	—	—	Moderate (++)—Indicates potent aqueous‐phase antioxidant action [178]

*Note:* (+++) strong consistent evidence, (++) moderate evidence, (+) limited evidence, (+/−) mixed/inconclusive evidence.

### 7.10. Neoxanthin

Neoxanthin is an epoxy‐containing xanthophyll carotenoid composed of carbon, hydrogen, and oxygen atoms. Among its isomers, 9 ^′^‐cis‐neoxanthin is a key pigment in photosynthetic organisms, residing within thylakoid membranes and associating closely with light‐harvesting complex II (LHCII). Each LHCII typically binds two luteins, one violaxanthin, and one 9 ^′^‐cis‐neoxanthin, where the latter contributes not only to light harvesting but also to photoprotective energy dissipation in conditions of excess light. In green algae such as *Chlamydomonas reinhardtii*, neoxanthin is naturally produced and functions as both an accessory pigment and a photoprotective factor [186].

In addition to its role in photosynthesis, neoxanthin exhibits strong antioxidant activity. Studies have shown that it prevents oxidative DNA damage more effectively than lutein, both in cell‐free assays and in *Chlamydomonas reinhardtii* extracts. Interestingly, combinations of neoxanthin and lutein led to reduced protection, likely due to competitive antagonism between the two xanthophylls [187]. Mechanistically, neoxanthin has been shown to suppress superoxide radical formation, inhibit the binding of carcinogens such as DMBA to DNA, and downregulate enzymes like ornithine decarboxylase, thereby interfering with both the initiation and promotion stages of carcinogenesis in animal models [188]. Furthermore, in human prostate cancer cells (PC‐3), neoxanthin induces apoptosis via caspase‐3 activation and poly (ADP‐ribose) polymerase cleavage, alongside modulation of Bax and Bcl‐2 expression, demonstrating direct antitumor potential [189]. Protective effects have also been reported in noncancerous cells, where neoxanthin reduces H_2_O_2_‐induced oxidative stress and prevents apoptosis by activating endogenous antioxidant defenses and preserving p53 activity in stress hormone–challenged keratinocytes [190].

Structurally, neoxanthin exists in all‐trans and 9 ^′^‐cis isomeric forms, both of which display distinctive absorption spectra due to the presence of a 5,6‐epoxide group, an allenic bond, and hydroxyl moieties. While it does not participate directly in the canonical xanthophyll cycle, it contributes to photoprotection by dissipating excess energy in species lacking a functional cycle, thereby reducing photoinhibition under high‐light stress [190].

Despite these promising biological activities, neoxanthin shows very low bioavailability in humans. A dietary intervention with fresh spinach (100 g/day for 4 weeks) significantly increased plasma lutein and *β*‐carotene but left neoxanthin undetectable, highlighting its poor systemic uptake [191]. By contrast, in mice, dietary neoxanthin reached plasma levels comparable to other carotenoids such as lutein and *β*‐carotene within hours of ingestion [191]. In vitro Caco‐2 assays similarly reflect this discrepancy, showing that neoxanthin uptake occurs at only ~25% the efficiency of lutein, reflecting a relative barrier to intestinal absorption [192]. The limited human bioavailability can be explained by several factors: (i) poor release from complex food matrices, particularly leafy vegetables rich in fibers and divalent minerals; (ii) limited passive diffusion due to its polarity and epoxide moiety; (iii) potential efflux by multidrug resistance transporters; and (iv) rapid conversion to neochrome and subsequent clearance, akin to the short plasma half‐lives observed for other epoxy‐xanthophylls like fucoxanthin [191]. An evidence grid for neoxanthin (Table 11) stratifies research findings by methodology, revealing the current state of translational progress.

**Table 11:** Evidence summary for neoxanthin health outcomes.

Outcome	In vitro evidence	Animal studies	Human studies	Strength of evidence and translational relevance
Photosynthetic role	Key LHCII pigment (9 ^′^‐cis‐neoxanthin); photoprotection/energy dissipation [186]	—	—	Moderate (++)—Well‐defined in photosystems [186]
Antioxidant and anticarcinogenic mechanisms	Prevents oxidative DNA damage (greater than lutein in assays); suppresses superoxide; inhibits DMBA‐DNA binding; ↓ ODC [187, 188]; induces apoptosis in PC‐3 via caspase‐3/PARP, Bax/Bcl‐2 modulation [189]; protects keratinocytes vs. H_2_O_2_/stress hormones via endogenous defenses and p53 preservation [190]	Interferes with initiation/promotion stages in animal carcinogenesis [189]	—	Moderate (++)—Strong cellular/animal support; no clinical data [187–190]
Isomeric/structural features	All‐trans and 9 ^′^‐cis forms; epoxide + allenic + hydroxyl groups; photoprotection outside canonical xanthophyll cycle [190]	—	—	Limited (+)—Structure–function insights support activity [177]
Bioavailability	Human: Very low—Spinach (100 g/day × 4 weeks) ↑ lutein/*β*‐carotene but neoxanthin undetectable [191]; mouse: Reaches plasma rapidly [191]; Caco‐2 uptake ~25% of lutein [192]	Matches higher uptake in mice [191]	—	Limited (+)—Significant human uptake barrier [191, 192]

*Note:* (+++) strong consistent evidence, (++) moderate evidence, (+) limited evidence, (+/−) mixed/inconclusive evidence.

### 7.11. Spirilloxanthin

Spirilloxanthin, a methoxy‐containing carotenoid predominant in many purple phototrophic bacteria (e.g., *Rhodoplanes roseus* and *Rhodoplanes serenus*), also exists in a potent antioxidant derivative, 3,3,4‐tetrahydrospirilloxanthin‐20‐al, isolated from *Rhodobacter sphaeroides* [156, 193].

Biochemically, spirilloxanthin demonstrates robust free radical–scavenging capacity on par with lycopene and exceeding *β*‐carotene, making it a highly effective quencher of ROS [194]. In its native phototrophic context, it plays a dual role as an accessory pigment, absorbing light within the 450–550 nm range, and as a safeguard for the photosynthetic machinery; it dissipates energy and quenches singlet oxygen, thereby protecting light‐harvesting complexes from oxidative stress [195, 196]. This photoprotective mechanism stems from its extensive conjugated system, which rapidly neutralizes excited chlorophyll states and ROS within the LH2 complexes, even when spirilloxanthin synthesis is genetically engineered into alternative bacterial systems [195]. While direct human bioavailability data for spirilloxanthin are lacking, general carotenoid research suggests that its lipophilic character and microbial origin may limit systemic uptake unless advanced extraction and delivery systems such as nanoemulsions or supramolecular carriers are employed. Encouragingly, such technologies have notably enhanced the bioavailability of related carotenoids and could offer a future path toward effective spirilloxanthin utilization in nutraceutical or therapeutic contexts [196]. Table 12 summarizes spirilloxanthin bioactivity data across multiple research domains, providing readers with a framework to assess evidence strength and clinical applicability.

**Table 12:** Evidence summary for spirilloxanthin health outcomes.

Outcome	In vitro evidence	Animal studies	Human studies	Strength of evidence and translational relevance
Sources and derivatives	Predominant in purple phototrophs; antioxidant derivative 3,3,4‐tetrahydrospirilloxanthin‐20‐al from *R. sphaeroides* [156, 192]	—	—	Limited (+)—Specialized microbial sources [156, 192]
Antioxidant and photoprotection	ROS‐quenching comparable to lycopene and > *β*‐carotene [193]; absorbs 450–550 nm; protects LH complexes; quenches singlet O_2_; engineered into LH2 complexes for protection [194, 195]	Photoprotection demonstrated in bacterial systems [194, 195]	—	Moderate (++)—Strong photoprotective capacity; translational data lacking [193, 195]
Bioavailability/delivery	—	—	Human data lacking: nanoemulsions/supramolecular carriers may be required, extrapolating from related carotenoids [195, 196]	Limited (+)—Tech pathways plausible; human PK unknown [195, 196]

*Note:* (+++) strong consistent evidence, (++) moderate evidence, (+) limited evidence, (+/−) mixed/inconclusive evidence.

## 8. Future Perspective

Marine carotenoids hold great promise as compounds for managing human health in the future, and they have been shown to have several biological and immunological activities. Astaxanthin and canthaxanthin showed antioxidant, anticancer, and anti‐inflammatory properties; can decrease the production of IL‐1, IL‐6, and TNF‐*α* cytokines; and enhance the activity of glutathione peroxidase and catalase. As a result, these carotenoids are recognized as neuroprotective agents for neurodegenerative diseases. Fucoxanthin has strong anti‐inflammatory properties by effectively inhibiting the NF‐*κ*B pathways that are activated by LPS. Furthermore, lutein has the capacity to eliminate ROS that are generated during inflammation and inhibit the generation of TNF‐*α* in cultivated ECs. In addition, research has shown that *β*‐carotene inhibits the movement of the NF‐*κ*B p65 subunit into the nucleus, thus preventing the phosphorylation and breakdown of the NF‐*κ*B inhibitor. Carotenoids are well recognized for their antioxidant activities, including their ability to inhibit free radical activity, mitigate damage caused by ROS, and prevent lipid peroxidation. These compounds have significantly influenced the control and enhancement of the immune system in vertebrates. Nevertheless, dietary carotenoids must take into account some vital factors to effectively prevent or cure a variety of diseases. The effectiveness of a certain carotenoid may change based on the levels of other carotenoids. Carotenoids exhibit a synergistic effect, rendering their combination into a supplement ineffective. Additionally, they are highly responsive to oxidative, chemical, or enzymatic processes, resulting in the synthesis of other substances with uncertain effects. Furthermore, individuals with comparable dietary intakes may respond differently to carotenoids due to their genetic susceptibility. Lastly, the impact of carotenoids is dependent on the dosage and duration of exposure. Further investigation is necessary to clarify the molecular biological properties, safety, and metabolism of carotenoids prior to their use in cancer prevention.

## 9. Limitations and Contradictory Findings

While numerous studies demonstrate the potent antioxidant and anti‐inflammatory effects of marine carotenoids in vitro and in animal models, the translation to human subjects remains a significant challenge. Controlled human clinical trials are sparse and often yield inconsistent results, highlighting the need for more rigorous and well‐designed studies. For example, some studies suggest that *β*‐carotene, at high concentrations or under specific conditions, may act as a pro‐oxidant, complicating its therapeutic application. Similarly, lycopene has shown proinflammatory effects in some contexts, suggesting its bioactivity is highly dependent on dosage and physiological state. These contradictory findings underscore the complexity of carotenoid metabolism and the need for personalized approaches to supplementation.

Another key limitation is the poor and variable oral bioavailability of many marine carotenoids. Their absorption is influenced by a multitude of factors, including the food matrix, dietary fat content, and individual genetic variations. For instance, a polymorphism in the *BCO1* gene can reduce lycopene cleavage activity, leading to higher plasma levels but potentially lower formation of bioactive metabolites.

Furthermore, the antimicrobial activities of many marine carotenoids are typically observed only at high in vitro concentrations, which may not be achievable in vivo, thus limiting their clinical relevance. These critical points must be considered to guide future research toward dose optimization and the development of validated delivery systems, such as nanoemulsions, to improve bioavailability.

Finally, regulatory and safety concerns vary by jurisdiction, and extraction methods lack standardization. These limitations must temper conclusions and guide future research toward well‐designed human studies, dose optimization, and validated formulations.

## 10. Conclusions

Marine carotenoids represent a promising class of compounds with significant potential for improving human health, as evidenced by their diverse biological activities. This review highlights their roles as potent antioxidants, anti‐inflammatory agents, and modulators of key cellular pathways. While several species have demonstrated therapeutic potential, particularly astaxanthin and fucoxanthin, a critical analysis reveals a number of research gaps that must be addressed to facilitate their clinical application.

The most significant research gaps include the need for well‐controlled, long‐term human clinical trials to validate the efficacy and optimal dosages observed in preclinical studies. Future research should also focus on elucidating the complex pharmacokinetics of these compounds and addressing their often poor oral bioavailability through advanced delivery systems such as nanoencapsulation. There is also a need for standardized extraction and purification methods to ensure product consistency and regulatory compliance.

Astaxanthin and fucoxanthin show the most immediate promise for future clinical application due to the extensive body of research supporting their anti‐inflammatory, antioxidant, and antiobesity effects. Astaxanthin, in particular, has a well‐established safety profile and has shown promise in managing chronic diseases. However, safety and regulatory issues, particularly for novel marine carotenoids, require thorough investigation. As the field advances, multidisciplinary approaches, including multiomics and systems biology, will be essential to fully elucidate the mechanisms of action and unlock the full potential of these marine‐derived bioactives.

## Conflicts of Interest

The authors declare no conflicts of interest.

## Author Contributions

**Gamal M. El-Sherbiny:** writing – review and editing, writing – original draft, conceptualization. **Mohamed H. Kalaba:** writing – review and editing, writing – original draft, conceptualization.

## Funding

No funding was received for this manuscript.

## Data Availability

The data associated with this study are included within the article.
